# Improving Nitrogen Use Efficiency Through Overexpression of Alanine Aminotransferase in Rice, Wheat, and Barley

**DOI:** 10.3389/fpls.2021.628521

**Published:** 2021-01-28

**Authors:** Jingwen Tiong, Niharika Sharma, Ramya Sampath, Nenah MacKenzie, Sayuri Watanabe, Claire Metot, Zhongjin Lu, Wayne Skinner, Yingzhi Lu, Jean Kridl, Ute Baumann, Sigrid Heuer, Brent Kaiser, Mamoru Okamoto

**Affiliations:** ^1^School of Agriculture, Food and Wine, University of Adelaide, Glen Osmond, SA, Australia; ^2^NSW Department of Primary Industries, Orange, NSW, Australia; ^3^ARC Industrial Transformation Research Hub for Wheat in a Hot and Dry Climate, Waite Research Institute, University of Adelaide, Glen Osmond, SA, Australia; ^4^Arcadia Biosciences, Davis, CA, United States; ^5^Rothamsted Research, Harpenden, United Kingdom; ^6^Centre for Carbon, Water and Food, University of Sydney, Brownlow Hill, NSW, Australia

**Keywords:** alanine aminotransferase, nitrogen use efficiency, transgenic cereals, RNAseq, carbohydrate metabolism

## Abstract

Nitrogen is an essential nutrient for plants, but crop plants are inefficient in the acquisition and utilization of applied nitrogen. This often results in producers over applying nitrogen fertilizers, which can negatively impact the environment. The development of crop plants with more efficient nitrogen usage is, therefore, an important research goal in achieving greater agricultural sustainability. We utilized genetically modified rice lines over-expressing a barley alanine aminotransferase (*HvAlaAT*) to help characterize pathways which lead to more efficient use of nitrogen. Under the control of a stress-inducible promoter *OsAnt1*, *OsAnt1:HvAlaAT* lines have increased above-ground biomass with little change to both nitrate and ammonium uptake rates. Based on metabolic profiles, carbon metabolites, particularly those involved in glycolysis and the tricarboxylic acid (TCA) cycle, were significantly altered in roots of *OsAnt1:HvAlaAT* lines, suggesting higher metabolic turnover. Moreover, transcriptomic data revealed that genes involved in glycolysis and TCA cycle were upregulated. These observations suggest that higher activity of these two processes could result in higher energy production, driving higher nitrogen assimilation, consequently increasing biomass production. Other potential mechanisms contributing to a nitrogen-use efficient phenotype include involvements of phytohormonal responses and an alteration in secondary metabolism. We also conducted basic growth studies to evaluate the effect of the *OsAnt1:HvAlaAT* transgene in barley and wheat, which the transgenic crop plants increased seed production under controlled environmental conditions. This study provides comprehensive profiling of genetic and metabolic responses to the over-expression of *AlaAT* and unravels several components and pathways which contribute to its nitrogen-use efficient phenotype.

## Introduction

Nitrogen (N) is an absolute requirement for plant growth and reproduction. Therefore, applying N fertilizer into cropping systems is an essential practice to secure productivity. Global N fertilizer consumption is more than 110 Mt per annum with half of the total being used for the production of major cereal crops (i.e., maize, rice, and wheat) (Ladha et al., 2016). Although there have been continuous improvements of N use efficiency (NUE) of crops over the years along with increases in crop yield (Ortiz-Monasterio et al., 1997; Ciampitti and Vyn, 2012; Sadras and Lawson, 2013), more than 50% of applied N fertilizers are unused by crops at the global scale (Raun and Johnson, 1999; Lassaletta et al., 2014). Unabsorbed N fertilizer in soil is an environmental concern due to leaching and atmospheric release through volatilization (Vitousek et al., 1997). Further improvement of NUE in crops is thus an important aim in agriculture research and our future food production capabilities.

Genotypic variation for NUE traits exists and has spurred breeding activities to develop N use-efficient crops. Examples of such genetic variation for NUE in cereal crops include N accumulation in rice (Borrell et al., 1998) and wheat (Le Gouis et al., 2000), N remobilization in maize (Hirel et al., 2007), and yield under low N (Le Gouis et al., 2000). It is expected that further extension of the germplasm pool through introgression of landraces and ancestral germplasm will help breeding programs which drive future NUE-based outcomes (Hawkesford, 2014). A recurrent difficulty is to identify the genetic control linked to NUE phenotypes observed in the field. This is due to the complexity of N metabolism during plant growth stages and the influence of environmental factors (DoVale et al., 2012).

There have been several studies attempting to improve NUE *via* genetic engineering. Obvious candidate genes of interest include the N transporters such as nitrate transporters (NRT) and ammonium transporters (AMT). Overexpression of *OsAMT1;1* in rice failed to improve growth at low N and showed variability in ammonium transport (Kumar et al., 2006). More recently, Fan et al. (2016) showed an enhanced NUE (∼40%) phenotype in rice through the overexpression of the nitrate transporter, *OsNRT2.3b* when grown under adequate N. Some improvements in NUE were observed by manipulating genes involved in N assimilation and regulation. These examples include glutamine synthetase, glutamate synthase, amino acid biosynthesis (such as alanine aminotransferase or asparagine synthetase), transcriptional regulators such as Dof1 (Kurai et al., 2011; McAllister et al., 2012), and autophagy genes such as *ATG8* (Yu et al., 2019; Fan et al., 2020a). Many of these studies were complemented with genetic (transcript) and/or biochemical profiling to study the physiological effects of manipulating these genes. Information obtained from these approaches can help decipher the role of a gene of interest in conferring an observed phenotype. In particular, omics technologies (e.g., transcriptomics, metabolomics, and proteomics) are powerful tools not only to characterize the plants of interest, but it could also help identify candidate genes for improving NUE and other traits (Hirai et al., 2004; Avice and Etienne, 2014; Mosleth et al., 2015; Tiwari et al., 2020a, b, c).

In this present study, we conducted an in-depth physiological and genetic profiling of rice lines over-expressing the barley *AlaAT* gene, driven by the rice antiquitin (*OsAnt1*) promoter (*OsAnt1:HvAlaAT*). AlaAT catalyzes the reversible conversion of glutamate and pyruvate to alanine and α-ketoglutarate, and alanine can be a source of amino acid during hypoxia (Good and Muench, 1993; Muench and Good, 1994; Miyashita et al., 2007). Previously, AlaAT has been tested as a candidate to improve NUE by targeted over-expression in the roots. Canola lines overexpressing *HvAlaAT* (driven by the root-specific *btg26* promoter) increased above-ground biomass production and yielded more seed under various N levels (Good et al., 2007). Rice lines over-expressing *HvAlaAT*, driven by the *OsAnt1* promoter, displayed higher root and shoot biomass production (Shrawat et al., 2008; Beatty et al., 2013; Selvaraj et al., 2017). In these latter works, metabolic profiling yielded inconclusive results, and transcriptome analysis did not indicate a change in N transport and assimilation. Transgenic sugarcane and wheat plants with *OsAnt1:HvAlaAT* also showed improved NUE and/or biomass production (Snyman et al., 2015; Peña et al., 2017). Our current study is an extension of the previous works in rice as mentioned above, including additional physiological analyses on N influx, and in-depth metabolomics and transcriptomic profiling. We also investigated the gene technology in barley and wheat.

## Materials and Methods

### Plant Materials

*OsAnt1:HvAlaAT* transgenic plants were produced in japonica rice (*Oryza sativa*, cv. Nipponbare) as described in Shrawat et al. (2008). Two events containing a single copy of the transgene, determined by quantitative PCR (Supplementary Figure S8A), were used for subsequent analyses. RNA expression was demonstrated (Supplementary Figure S10A) as well as increased AlaAT protein (Supplementary Figure S8B; Skinner et al., 2012) and enzymatic activity (Supplementary Figure S9). The transgene was transformed into barley (*Hordeum vulgare*, cv. Golden Promise) using *Agrobacterium*-mediated transformation and the method developed by Tingay et al. (1997) and modified by Matthews et al. (2001). Wheat (*Triticum aestivum*, cv. Gladius) was transformed using microprojectile bombardment as described by Kovalchuk et al. (2009). The list of all experiments performed in this study is presented in Supplementary Table S1.

### Rice Field Trial

Confined field trials were conducted in Five Points, California, from May to October, in 2008 and 2009 in flooded basins, under both notifications by the USDA-APHIS and permits by the California Rice Commission (CRC). Rice seedlings (Westside Transplants, Huron, CA, United States) were transplanted at 3–4 leaf stage into flooded basins and supplemented with different levels of N at several growth stages. Plots were 1 m × 4 m in size in a split-plot design with three replicates per genotype. Plants were 10 cm apart with 50 cm between plots. N was applied in the form of urea in three splits (e.g., for a total of 123 kg ha^–1^: (1) 45 kg ha^–1^ at basin preparation, (2) 33 kg ha^–1^ 2 weeks after transplanting, and (3) 45 kg ha^–1^ at flowering). Water levels were maintained to flood the basins throughout the trial until 4 weeks before harvest. Crops were harvested, and seed yield was determined at grain moisture of 12%.

### Plant Growth in Ebb and Flow Hydroponic System in Growth Room

Rice seeds were dehusked, surface-sterilized and imbibed in Petri dishes on a filter paper with sterilized deionized water. Seeds were incubated in a growth chamber (100–130 μmol m^–2^s^–1^, cycle of 12/12 h light/dark, 28°C) for 10 days. Uniformly germinated seedlings were then transferred to one of two 700 L ebb and flow hydroponic systems (Garnett et al., 2013), with a complete fill/drain cycle of 15 min (two separate systems for each N treatment). Each system contained 100 plants (20 plants per genotype; five genotypes which included two independent transgenic lines, nulls, and wildtype. Individual seedlings were grown on mesh collars within tubes (300 mm × 50 mm). The nutrient solution was a modified Johnson’s solution (Johnson et al., 1957) containing (in mM), 0.5 N (9 units NO_3_^–^:1 unit NH_4_^+^), K:2.95, Ca:1.25, Mg:0.5, S:1.25 and P:1 for the 0.5 mM N treatment, and 2.5 N (9 units NO_3_^–^:1 unit NH_4_^+^), K:3.05, Ca:1.75, Mg:0.5, S:0.5, P:1 for the 2.5 mM N treatment. Both treatment solutions also contained (in μM): Mn:2, Zn:2, B:25, Cu:0.5, Mo:0.5, Fe:100 (as Fe-EDTA). The hydroponic system was situated in a controlled environment room with a day/night cycle of 14/10 h, 26/20°C, with a flux density at the canopy level of *c.* 650 μmol m^–2^s^–1^ and relative humidity of 60%. Solutions were maintained between 19 and 21°C. Solution pH was maintained between 5.8 and 6.2 using CaCO_3_ and changed every 7 days. After grown 6 weeks in the system, the plants were subjected to study for N fluxes, metabolic profiling, and transcriptome analysis.

### Whole Season Rice Growth in Glasshouse

Seedlings were prepared as above and placed in square pots (13 cm × 13 cm) containing diatomaceous stones. The pots were placed in black trays (40 plants/tray) with a continuous fill/drain cycle hydroponics system using the same nutrient solution composition as above (2.5 mM N), with the solution replaced every 10 days. Plants were grown during a summer growth season (2013/2014) in a glasshouse with a day/night temperature of 28/20°C and relative humidity of approximately 65%. Shoots and grains were harvested at maturity and oven-dried at 80°C for 48 h.

### Barley and Wheat Growth in Glasshouse

Barley lines were grown in pots (15 cm in diameter and height) containing coco peat potting mix (Tiong et al., 2014) in a single N treatment (110 mg kg^–1^ soil from Ca(NO_3_)_2_, plus 400 mg kg^–1^ soil from slow-release Osmocote^®^ fertilizer). Three independent transgenic lines were used, along with nulls and wildtype, with ten replicates per line. Wheat lines were grown in soil bins (W110 × D90 × H70 cm) consisting of 700 kg soil mix (1:1 ratio of cocopeat-mix:UC Davis mix, Nauer et al., 1967). Plants were grown in a glasshouse with approximately 24/13°C day/night temperature. Two N treatments were used: N40 (40 kg N ha^–1^) and N80 (80 kg N ha^–1^) applied as urea at planting. Three independent transgenic lines were used for each N treatment, along with nulls and wildtype. Each line was grown in a row of 10 plants per replicate. We had four replicates, totaling to 40 plants per line with each N treatment. Reverse osmosis water was used for watering during the experiment. Shoots and grains were harvested at maturity and oven-dried at 80°C for 48 h.

### NO_3_^–^ and NH_4_^+^ Influx Measurement

On sampling days, between 11:00 and 13:00, plants from the ebb and flow system were transferred to nutrient solutions chemically identical to the ones they were grown in. Roots were then given a 1 min rinse with the same nutrient solution containing either 100 or 1000 μM NO_3_^–^ or NH_4_^+^, followed by 10 min of exposure to the same solution, but with ^15^N-labeled NO_3_^–^ or NH_4_^+^ (^15^N, 30 atom%). The concentration of 100 μM was used as it is thought to be close to saturation of the high-affinity transporter system (HATS) and 1000 μM would include both HATS and low-affinity transporter system (LATS) uptake (Siddiqi et al., 1990; Kronzucker et al., 1995; Crawford and Glass, 1998). At harvest, roots and shoots were separated, dried (5 days, 60°C), weighed and ground. ^15^N content of dried plant samples was determined using an EA-IRMS (University of California, Davis Stable Isotope Facility). Mean LATS influx values were calculated by subtracting the mean 100 μM influx value from that of 1000 μM at the same time-point and treatment (Okamoto et al., 2003).

### Tissue NO_3_^–^ Determination

NO_3_^–^ was extracted from 5 mg of homogenous finely-ground freeze-dried plant tissue added to 1 ml MilliQ-H_2_O and boiled in a water bath (20 min, 95–100°C). Nitrate was measured by the Cataldo’s method (Cataldo et al., 1975) where the complex formed by nitration of salicylic acid under highly acidic conditions was measured at 410 nm in basic solutions.

### Tissue NH_4_^+^ Determination

NH_4_^+^ was extracted from 10 mg of homogenous finely-ground freeze-dried plant tissue added to 1 ml MilliQ-H_2_O and mixed vigorously for 30 min. The filtered extracts were then measured for NH_4_^+^ by a phenol-hypochlorite method for determining ammonia in water using nitroprusside as catalyst (Solórzano, 1969).

### RNA Isolation, RNA Sequencing, and Quantitative Real-Time RT-PCR

Briefly, total RNA from roots and shoots of rice was prepared using TRIzol^TM^ reagent according to the manufacturer’s instructions (Invitrogen) and treated with DNase I (Ambion). The RNA was used for RNA-Seq (100 ng RNA per sample), and for QRT-PCR. For RNA-Seq, RNA from four biological replicates of roots and shoots of one transgenic, *OsAnt1:HvAlaAT* overexpressing event, and two biological replicates of roots and shoots of the corresponding null and wildtype (spp. Nipponbare) were used (totaling to 16 samples). The non-stranded Illumina TruSeq libraries were prepared and run on a HiSeq 2500 to give 2 × 100 PE reads at Australian Cancer Research Foundation Cancer Genomics Facility. For QRT-PCR, two micrograms of total RNA were used to synthesize cDNA with SuperScript^TM^ III reverse transcriptase (Invitrogen). Three biological replicates were used for transcript analysis with three technical replicates for each cDNA sample. Normalization was carried out as described by Vandesompele et al. (2002). The normalized copies μg^–1^ RNA were used to represent transcript levels. The primer sequences for all genes analyzed are listed in Supplementary Table S2.

### Metabolite Quantification

Ground lyophilized rice shoot and root tissues were used for this analysis. Chemicals and metabolite standards were pure (≥98%) and were purchased from Alfa Aesar (Ward Hill, MA, United States), EMD Millipore (Billerica, MA, United States) or Sigma Aldrich (St Louis, MO, United States). Metabolites, including free amino acids, were extracted as described by Hacham et al. (2002). Tissue (35 mg leaf and 15 to 20 mg root) was extracted in 1 mL of 0.1 M HCl, containing 0.2 mg each of D7-Glucose (1,2,3,4,5,6,6-d7, 97 atom%, Sigma Aldrich), 0.2 mg of D7-L-Alanine [CD3CD(ND2)COOD, 98 atom%, CDN Isotopes, Point-Claire, Quebec] and D3-methionine (S-methyl-d3, 98 atom%, CDN Isotopes) as internal standards. Samples were periodically vortexed in the 0.1 N HCl solution at RT for 1 h followed by the addition of 1 mL methanol and 0.5 mL chloroform and extraction of metabolites by vortexing (three times for 10 s on high) before the two phases were separated by centrifugation (3,000 rpm, 15 min). The upper polar phase was dried under nitrogen and the dry residue derivatized with 100 μl of 20 mg mL^–1^ methoxylamine HCl in dry pyridine (50°C, 1 h), followed by 100 μL of MSTFA + 1% TMCS (50°C, 1 h) before analysis by full-scan GC/MS (Agilent 6890/5973i), as described by Roessner et al. (2000). Quantification of metabolites was based on internal standard calibration curves using standards at nine concentrations ranging from 1 to 100 μg (extracted and derivatized as above). Alanine and methionine were quantified using their heavy isotope internal standards while all other metabolites employed D7-Glucose as the internal standard. Duplicates of each sample were processed and analyzed whenever sufficient tissue was available.

The method precision and accuracy were tested using seven samples each of ground pooled wildtype rice leaf; leaf tissue spiked with 10 μg of each metabolite standard; and a mixture of all metabolite standards without tissue matrix. The average precision (RSD) for all metabolites was 18% for rice leaf, 11% for metabolite-spiked rice leaf, and 8% RSD for standards without matrix. The accuracy of the method using a mixture of all metabolite standards averaged 84.5%.

### Bioinformatics

#### Read Filtering, Mapping and Differential Expression Analysis

The raw data obtained were subjected to quality control using FastQC version 0.11.2^1^, and the reads were filtered out for mapping to organelle and rRNA sequences using Bowtie2 version 2.2.3 (Langmead and Salzberg, 2012). Rice chloroplast and mitochondrial sequences were downloaded from ftp://ftp.plantbiology.msu.edu/pub/data/Eukaryotic_Projects/o_sativa/annotation_dbs/pseudomolecules/ and rRNA sequences were from the NCBI nucleotide database http://www.ncbi.nlm.nih.gov/. Bowtie indices were constructed with the “bowtie2-build” command using – offrate 1, –large-index parameters from the sequence fasta files. Ungapped alignments were performed, and reads which were mapped with ≤2 mismatches were thrown out. Other parameters used for Bowtie2 were –N 1, -L 10, -i S,1,0.25, -D 30, -R 4, –mp 2, –np 2, –ma 0 and –score-min L,4,0.

The reads were mapped to the *O. sativa* subsp. *japonica* reference genome (build MSU7.0) ftp://ftp.plantbiology.msu.edu/pub/data/Eukaryotic_Projects/o_sativa/annotation_dbs/pseudomolecules/version_7.0/all.dir/ using TopHat version 2.1.0 (Trapnell et al., 2009). This software requires Bowtie2 and SAMTools (Li et al., 2009) downloaded from http://samtools.sourceforge.net/version 0.1.19. The reference sequence file and the gff3 files were added with the sequence of *OsAnt1:HvAlaAT*, and Bowtie2 index was built using “bowtie2-build”command and the same parameters as described above. Further standard TopHat parameters with –G option were used with the exception of the following options: -N 1, –read-gap-length 0, –read-edit-dist 1, –no-discordant, –no-mixed, –b2-rdg 999999, –b2-rfg 999999, –b2-N 1, –b2-L 10, –b2-i S,1,0.25, –b2-D 30, –b2-R 4, –b2-mp 2,2, –b2-np 2, –b2-score-min L,-2,0, -i 16, and -I 20000. All sixteen samples were mapped separately using the same TopHat settings. Finally, after running Cuffcompare, Cuffdiff program was run for roots and shoots samples with –b option. This methodology was called differential gene expression analysis without gene and transcript discovery.

#### Transcription Factor (TF) Encoding Genes

All TFs for *Oryza sativa cv. japonica* were downloaded from http://plntfdb.bio.uni-potsdam.de/v3.0/downloads.php?sp_id=OSAJ. The differentially expressed genes from Cuffdiff output in roots and shoots tissue samples were assigned to different TF families according to the Plant Transcription Factor Database assignments^2^. Expression information for these genes was from the Cuffdiff output.

#### Gene Ontology Enrichment Analysis

Gene Ontology enrichment of differentially expressed genes was performed using BiNGO tool (Maere et al., 2005). The metabolic pathway data available in the RiceCyc database of Gramene (Jaiswal et al., 2006) were analyzed to identify the enriched metabolic pathways in various gene sets. Both the GO and pathway enrichment analyses were performed at *p*-value cut-off of ≤0.05 after applying Benjamini-Hochberg correction.

## Results

### Phenotypic Assessment of Growth and Yield: *OsAnt1:HvAlaAT*-Transformed Rice, Barley and Wheat Lines

**FIGURE 1:**
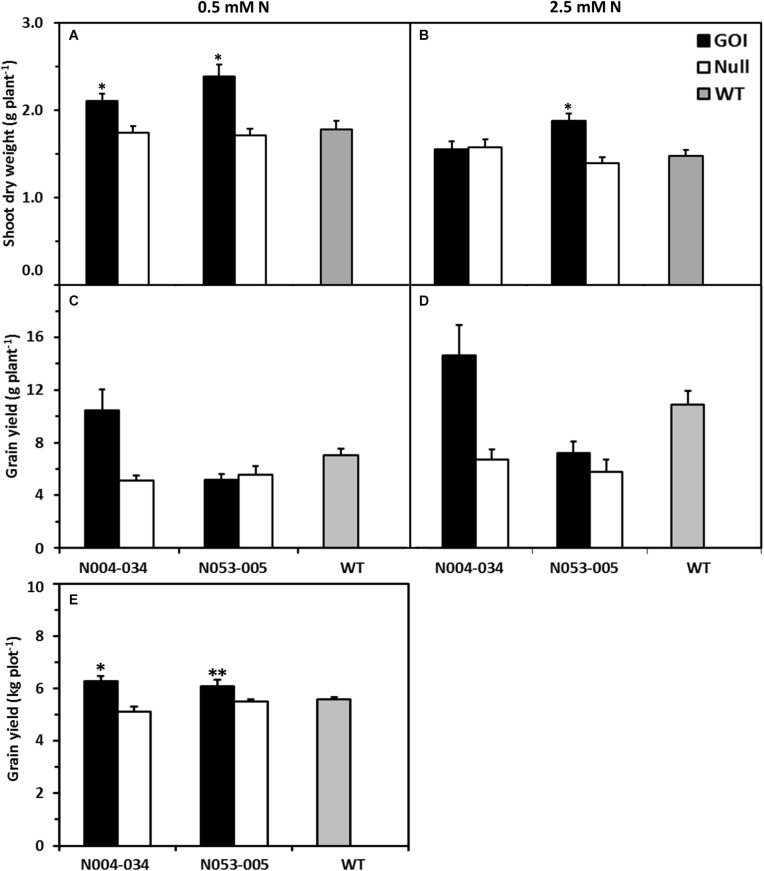
Shoot biomass and grain yield of rice plants expressing *OsAnt1:HvAlaAT* grown in hydroponics and field (grain yield only for the latter). Two T9 independent homozygous lines (Gene of Interest, GOI, black bars), their corresponding nulls (white bars) and wildtype (WT, gray bars) were assessed for biomass and grain yield. Plants were grown in an ebb and flow hydroponic system under 0.5 mM **(A)** and 2.5 mM N **(B)** for 42 days for vegetative shoot biomass **(A,B)**, and grown to maturity in a fill/drain cycle hydroponic system under 0.5 mM **(C)** and 2.5 mM N **(D)** for grain yield **(C,D)**. Means and SE values (error bars) of five replicates are presented, while asterisks indicate the least significant difference at *P* < 0.05 against both null and WT^∗^. These lines were also field tested (Five Points, California, 2009) under limiting N **(E)**. In total, 123 kg N ha^– 1^ was supplied throughout the season, corresponding to approximately 70% of typical N rates for rice in California. Means and SE values (error bars) of three replicates are presented, while asterisks indicate least significant difference at *P* < 0.05 against both null and WT^∗^ or against null only^∗∗^.

Two independent transgenic homozygous *OsAnt1:HvAlaAT* rice lines in *Oryza sativa*, cv. Nipponbare background (henceforth known as N004-034 and N053-005), along with corresponding nulls and wildtype (non-transformed) control were assessed for growth parameters in two hydroponics systems: (1) a tank-based ebb and flow recirculation system located inside a temperature-controlled growth chamber and (2) a continuous fill/drain cycle system inside a glasshouse with natural and supplemented lighting. After 42 days of growth (52 days after seed imbibition, DAI) in the ebb and flow system with 0.5 mM N (low N) in the nutrient solution, both transgenic lines displayed higher shoot dry weight (DW) compared with nulls and wildtype (by 16–38%) (Figure 1A). However, under 2.5 mM N (high N), only line N053-005 displayed significantly higher shoot DW compared to both its null and wildtype by 33 and 27%, respectively (Figure 1B). There were no significant differences in root DW between all genotypes in both N treatments (Supplementary Figure S1).

The *OsAnt1:HvAlaAT* rice lines were also grown in 0.5 and 2.5 mM N (low and high N, respectively) for one growth season (2013/2014) in a continuous fill/drain cycle hydroponics system located in a glasshouse. Although not statistically significant, N004-034 consistently yielded more grain (Figures 1C,D). This was surprising, as N053-005 had higher shoot biomass than N004-034 at a vegetative stage in the previous growth chamber experiment (Figures 1A,B). This outcome may be explained by harvest index (ratio of grain yield to total above ground biomass). Although not statistically significant, N004-034 consistently showed a higher harvest index than all other genotypes (Supplementary Figure S2).

The *OsAnt1:HvAlaAT* rice lines (cv. Nipponbare) were also grown in the field for one season (May to October 2009 in Five Points, California) to assess grain yield performance in agricultural conditions under limiting N. Both transgenic lines displayed higher grain yield with N004-034 having significantly higher grain yield compared to its null and wildtype by 23 and 13%, respectively. For N053-005, grain yield was only significantly higher than its null by 11% (Figure 1E). Additionally, we assessed the grain yield performance of a single transgenic rice line in an *Oryza sativa*, cv. Taipei background and the transgenic line also showed 30% higher grain yield compared to wildtype in the field condition (Supplementary Figure S3).

**FIGURE 2:**
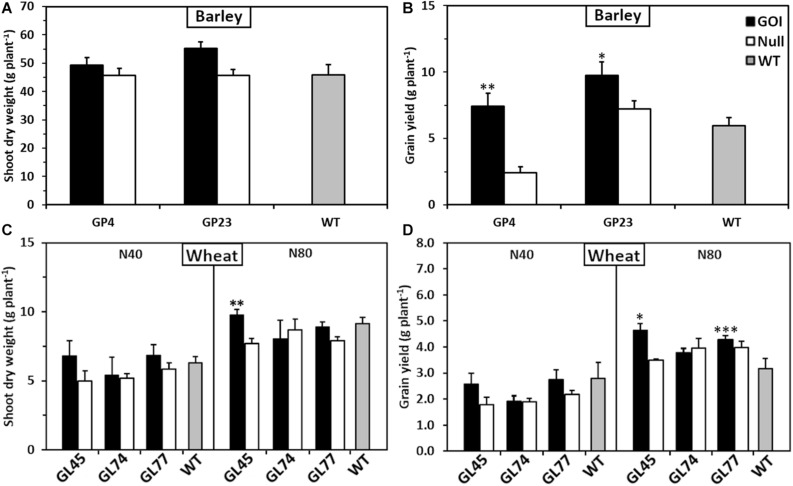
Shoot biomass and grain yield of glasshouse-grown barley and wheat plants expressing *OsAnt1:HvAlaAT*. Transgenic plants of three independent homozygous lines, null and wildtype were grown in a glasshouse. For barley **(A,B)**, T2 lines were grown in a single N treatment [110 mg as Ca(NO_3_)_2_, 400 mg as Osmocote N kg^– 1^ soil] in individual pots. For wheat **(C,D)**, T4 lines were grown in rows of ten in soil bins under two N treatments (N40, 40 kg N ha^– 1^; N80, 80 kg N ha^– 1^). Asterisks indicate least significant difference at *P* < 0.05 against null and WT^∗^, only against null^∗∗^, or only against WT^∗∗∗^. Means and SE values (error bars) of ten replicates are presented.

To determine the versatility of *OsAnt1:HvAlaAT* to improve NUE in other cereal crops, the construct was transformed into barley and wheat. Growth trials using homozygous (T2 barley, T4 wheat) lines were conducted in either soil pots (barley) or large soil bins (wheat) in a controlled-environment glasshouse. In both studies, there was evidence that selected *OsAnt1:HvAlaAT* expressing lines from both barley and wheat resulted in greater shoot biomass and grain yield compared to controls (Figure 2). For instance, two independent transgenic barley lines, GP4 and GP23, had significantly higher grain yield compared with their nulls by 226 and 37%, respectively. GP23 also had significantly higher grain yield than WT by 63% when grown under adequate N (only one N treatment was used for barley) (Figure 2B). In wheat, when grown under 80 kg N ha^–1^ (high N), line GL45 displayed significantly higher shoot biomass only against the null by 26% but had significantly higher grain yield compared to both null and wildtype by 32 and 47%, respectively (Figures 2C,D). Line GL77 displayed significantly higher grain yield only against WT by 36% when grown under high N (Figure 2D). These observations are reflective of results seen in rice (Figure 1).

### Nitrogen Uptake in *OsAnt1:HvAlaAT* Rice Lines

Further physiological and molecular characterizations were carried out with the rice lines. First, N influx studies were conducted with *OsAnt1:HvAlaAT* rice lines in *Oryza sativa*, cv. Nipponbare background grown hydroponically using an ebb and flow system at 0.5 or 2.5 mM N (low and high N, respectively). At 52 DAI, intact plants were subjected to separate treatments of either ^15^NO_3_^–^ or ^15^NH_4_^+^ at 100 or 1000 μM to measure the constitutive high-affinity transport system (HATS) or low-affinity transport system (LATS) uptake activities, respectively.

**FIGURE 3:**
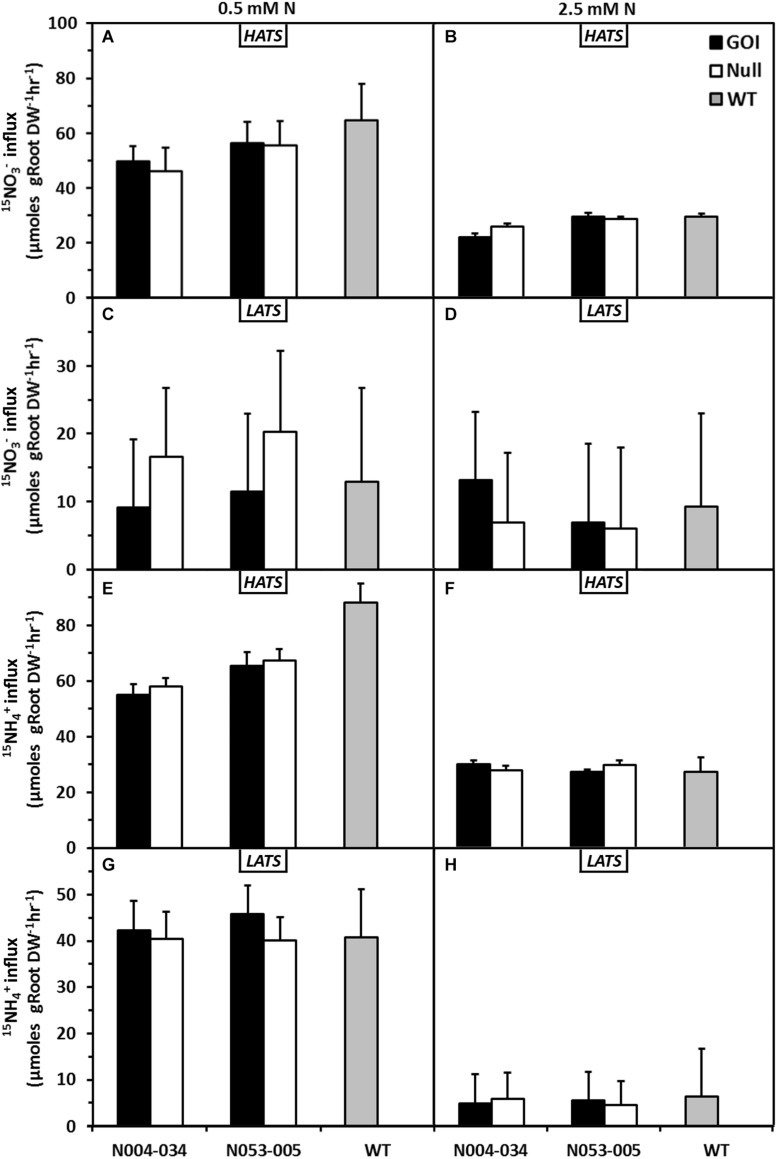
Effects of low and adequate N treatment on nitrate and ammonium uptake in rice plants via high- and low-affinity transport systems (HATS and LATS). Nitrate HATS influx **(A,B)** or LATS influx **(C,D)** and ammonium HATS influx **(E,F)** or LATS influx **(G,H)** of plants previously grown in 0.5 and 2.5 mM N. Transgenic plants of two independent lines, their corresponding nulls and wildtype were grown in a hydroponic system for 42 days (52 DAI) and subjected to uptake measurements using ^15^N labeled N sources at 100 μM (HATS) and 1000 μM (LATS). HATS values are means + SE (Standard Error, *n* = 5), whereas LATS are calculated means + SED (Standard Error of Difference between two means, *n* = 5).

In general, NO_3_^–^ influx into plants grown at low N was higher than those grown in high N (Figures 3A–D). This may be a response to an extended period of low N availability. For the transgenic lines, HATS NO_3_^–^ influx was similar to the nulls and wildtype control regardless of prior N treatments (Figures 3A,B). Correspondingly, the LATS NO_3_^–^ influx of both low and high N-grown transgenic lines were similar to the nulls and wildtype (Figures 3C,D). There were no differences in total NO_3_^–^ accumulation between all genotypes (Supplementary Figure S4).

With NH_4_^+^, there was an overall increase of influx (HATS and LATS) in plants previously grown at low N compared to those grown in high N (Figures 3E–H). Between genotypes, there was no difference in either HATS or LATS NH_4_^+^ influx, regardless of previous treatments (Figures 3E–H). As observed with the NO_3_^–^ influx studies, there was also no difference in total NH_4_^+^ accumulation between genotypes (Supplementary Figure S5).

Total N accumulation in plants grown in low N was not significantly different from that of plants grown in high N (Supplementary Figure S6). However, the shoot N accumulation of N005-035 was significantly higher than its null and wildtype previously grown under low N, but this trend was not observed when grown under high N. In summary, under the conditions examined in this study, there is little evidence to suggest directed overexpression of *HvAlaAT* had any impact on the rate of NO_3_^–^ or NH_4_^+^ uptake (HATS or LATS) into the hydroponically grown plants.

Another N influx study was conducted with *OsAnt1:HvAlaAT* rice lines in *Oryza sativa*, cv. Taipei background with the same setup as above using one independent transgenic line. Similar to the results observed in cv. Nipponbare background, the transgenic Taipei rice line displayed no differences in NO_3_^–^ or NH_4_^+^ uptake (HATS or LATS) compared to its null and WT (Supplementary Figure S7).

### Metabolic Profiling in *OsAnt1:HvAlaAT* Rice Lines

**FIGURE 4:**
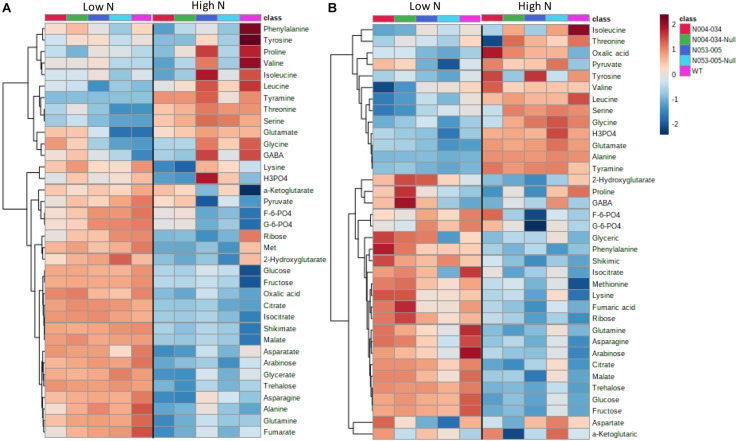
Metabolic heatmaps of rice plants expressing *OsAnt1:HvAlaAT*. Two transgenic rice lines, their corresponding nulls, and wildtype were grown hydroponically in low N (0.5 mM N) and high N (2.5 mM N) for 42 days. Metabolite levels of the shoot **(A)** and root **(B)** were processed by median normalization, log-transformed, and auto-scaling with MetaboAnalyst (Chong et al., 2019). The hierarchical clustering analysis was performed with Pearson’s distance measure.

Metabolic profiling by GC-MS was performed on *OsAnt1:HvAlaAT* rice lines’ root and shoot tissues from samples grown under 0.5 and 2.5 mM N (low and high N, respectively). The metabolite profiles highlighted many instances where both *OsAnt1:HvAlaAT* lines simultaneously displayed either significantly higher or lower concentration differences to wildtype (Figure 4 and Supplementary Tables S3A,B, S4A,B). However, this was not witnessed in comparison with nulls, whereby many metabolites were either higher or lower in one line compared to its null, but reversed in the other line (Supplementary Tables S3C,D, S4C,D). Furthermore, pyruvate was the only metabolite where, when grown under high N, both lines displayed a consistent difference (higher) compared to both their wildtype and nulls (only in roots; Supplementary Tables S4B,D).

In the comparison between the transgenic lines and wildtype under low N (0.5 mM), significant reduction in carbohydrates, namely glucose, fructose and arabinose, was observed in roots, while arabinose and ribose were lower in shoots (Figure 4 and Supplementary Tables S3A,B). Metabolites involved in the TCA cycle were altered in the transgenic lines compared to the wildtype in shoots, with citrate and isocitrate elevated and fumarate decreased (Figure 4A and Supplementary Table S3A). In addition, gamma aminobutyric acid (GABA) was increased while phosphoric acid was decreased in shoots. Although the sum of total free amino acids showed no difference, some amino acids displayed significant changes in shoots. For example, glutamate, glycine and threonine were elevated, while glutamine, asparagine and lysine were decreased (Figure 4A and Supplementary Table S3A). Similarly, asparagine was decreased in roots, while the other amino acids were unchanged in comparison to the wildtype (Figure 4B and Supplementary Table S3B).

Under high N (2.5 mM), the number of changes was reversed compared with low N, with shoots showing fewer changes than roots (Figure 4 and Supplementary Tables S4A,B). Only 2-hydroxyglutarate was decreased in both tissues (Figure 4 and Supplementary Tables S4A,B). In roots, leucine and isoleucine were decreased, while lysine and tyramine were elevated (Figure 4B and Supplementary Table S4B). Among the metabolites, oxalic acid and pyruvate were increased relative to wildtype (WT) (Figure 4B and Supplementary Table S4B).

Alanine was below the level of detection in the roots of low N-grown plants, while there was no difference in alanine concentration in *OsAnt1:HvAlaAT* lines compared to controls in either tissue under the high N treatment (Figure 4 and Supplementary Tables S3A,B, S4A,B). This is unexpected, as the overexpression of *AlaAT*, which did result in abundant AlaAT protein expression and enzyme activity (Supplementary Figures S8, S9 and Supplementary Method), would be expected to result in a higher rate of alanine production. However, as the reaction catalyzed by AlaAT is reversible, we may not be able to accurately predict the proportion of substrate/product based on enzyme activity alone.

### Deep Sequencing Transcriptomics of *OsAnt1:HvAlaAT* Rice Lines

#### Transcriptome Sequencing

Root and shoot samples of N053-005 grown under 0.5 mM N (low N), along with its null and wildtype, were used for RNA-Seq analysis. N053-005 was chosen on the basis that it demonstrated the clearest physiological difference compared to its controls (Figures 1A,B). We constructed non-stranded Illumina TruSeq libraries and generated a total of approximately 622 million paired-end sequence reads from 16 root and shoot samples (ranging from 28 to 47 million reads for each sample). The reads were filtered for organelle or rRNA sequences. The remaining reads were aligned to the rice reference genome using TopHat; approximately 80–84% of the reads were mapped.

#### Differential Gene Expression Analysis

**FIGURE 5:**
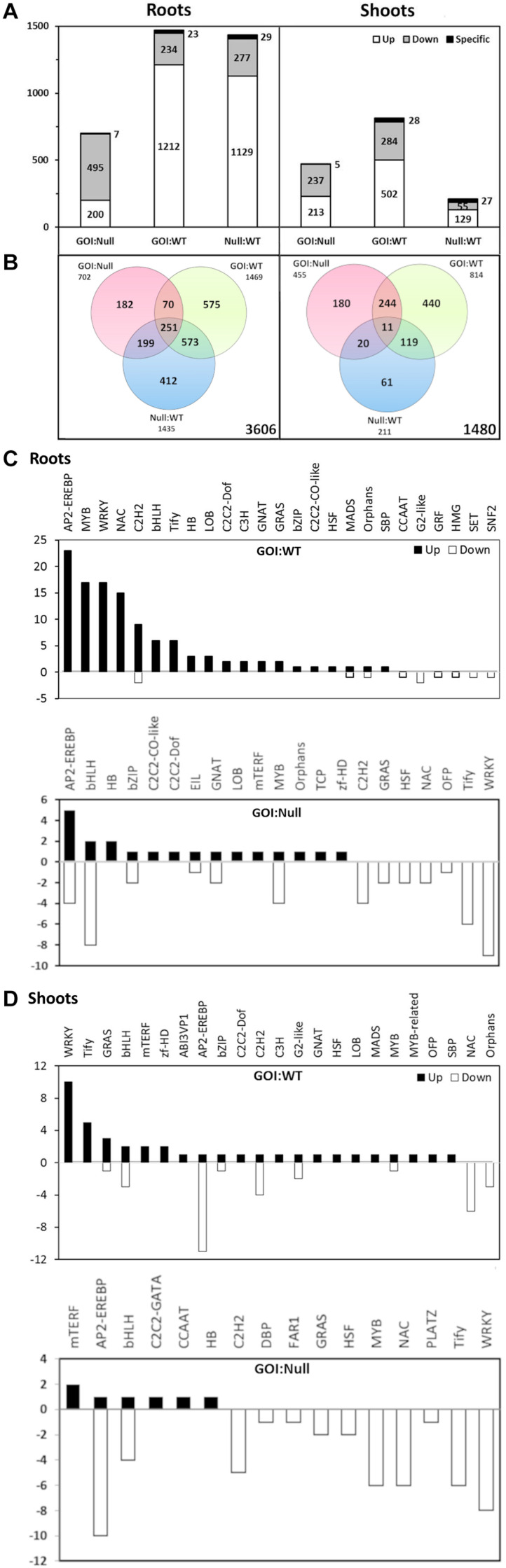
Differential gene expression of *OsAnt1:AlaAT* rice line N053-005 (GOI) compared with its null and wildtype (WT) in roots and shoots under 0.5 mM N **(A,B)**. The number of up- and down-regulated genes (white and gray bars, respectively) is shown in the bar graph **(A)**, with the number of specific transcripts (i.e., transcripts unique only to each comparison) being shown on top of each bar. The Venn diagram **(B)** shows the number of genes differentially expressed in each of the genotype comparisons. The numbers of differentially expressed transcripts between *OsAnt1:HvAlaAT* lines and wildtype/nulls representing different transcription factor families are shown in roots **(C)** and shoots **(D)**.

Differential gene expression analysis was performed using Cuffdiff, which resulted in 1469 differentially expressed genes (DEGs) in the gene of interest (GOI):WT comparison, while the GOI:Null and Null:WT comparisons had 702 and 1435 DEGs, respectively, in roots (Figures 5A,B). The ratio of DEGs that were upregulated in the roots of both GOI:WT and Null:WT comparisons far outweighs that of the downregulated ones, whereas an opposite trend was observed in the GOI:Null comparison (Figure 5A). In the shoots, the GOI:WT comparison shows the largest number of DEGs at 814. Meanwhile, the GOI:Null and Null:WT comparisons had 455 and 211 DEGs, respectively (Figures 5A,B). It is worth mentioning that the impact the transgene has on the overall transcriptome is minor; of the 66,124 total transcripts, the total number of DEGs in GOI:WT and GOI:Null comparisons in both roots and shoots accounts for only around 3 and 2%, respectively. The lists of DEGs for GOI:WT and GOI:Null comparisons in roots and shoots are present in Supplementary Tables S5–S8.

We are also interested in the DEGs which overlapped between the GOI:WT and GOI:Null comparisons (70 DEGs in roots, and 244 DEGs in shoots; Figure 5B). From the GO enrichment analysis of the overlapping DEGs, significant GO categories are represented by metabolic processes and stress response in both roots and shoots (Supplementary Tables S9, S10).

Expressions of genes associated with N uptake and assimilation were largely unaffected by the *AlaAT* transgene overexpression (Supplementary Tables S5–S8). Only five such genes showed changes in expression in the GOI:WT comparison: *OsAMT1.3* and *OsNRT2.1* were downregulated in the roots, *OsNRT1.2* was upregulated in the roots, and a putative nitrate reductase (LOC_Os02g53130) and a putative nitrite reductase (LOC_Os02g52730) were downregulated in the shoots and roots, respectively.

To validate the results from RNA-Seq, several differentially expressed genes reported previously (Shrawat et al., 2008; Beatty et al., 2013) were selected for quantitative RT PCR (QRT-PCR) analysis (Supplementary Figure S10 and Supplementary Table S2). The chosen genes include root-specific genes (*OsPRX20*, *OsGER2*, and *OsAMT1;3*; Druka et al., 2002; Passardi et al., 2004) and one shoot-specific gene (*OsRIR1a*; Mauch et al., 1998). The QRT-PCR analysis revealed a similar expression pattern to RNA-Seq for all selected genes (Supplementary Figure S10B). In the same experiment, we also included additional *AMT* (*OsAMT3;2* and *OsAMT1;1*) and *NRT* (*OsNRT2;2*, *OsNRT1;1*, and *OsNRT3;1*) genes, and there were no significant differences in the expression level of these N transporters in *OsAnt1:AlaAT* lines compared to wildtype (Supplementary Figure S10C).

#### Gene Ontology (GO) Enrichment Analysis of DEGs in GOI:WT and GOI:Null Comparisons

The rice transcripts were assigned GO terms under biological process, molecular function and cellular component categories. Among the differentially expressed biological process terms, metabolic processes were most represented in both roots and shoots (Supplementary Figures S11A,B). In the molecular function GO terms, the largest number of transcripts belonged to catalytic activity and binding in roots and shoots (Supplementary Figures S11A,B). Among the cellular component GO terms, the transcripts related to cell and membrane were the largest in number in both roots and shoots (Supplementary Figures S11A,B).

The BiNGO tool was also used to determine the enriched GO categories represented in the DEGs between the *OsAnt1:HvAlaAT* lines, wildtype and nulls in roots and shoots. Clearly, GO terms associated with various metabolic processes such as carbohydrate metabolism, chitin catabolism, and lipid biosynthesis/metabolism were significantly enriched in both tissues (Supplementary Figure S12). GO terms associated with nucleic acid (DNA and RNA) binding were also significantly enriched in roots of GOI:WT (Supplementary Figure S12A).

#### Transcription Factor Analysis

We further analyzed the rice sequencing data to identify the transcription factor (TF)-encoding genes. A total of 195 DEGs encoding TFs were identified in the GOI:WT comparison, with 124 in roots and 71 in shoots, whereas a total of 126 DEGs were identified in the GOI:Null comparison, with 67 in roots and 59 in shoots (Figures 5C,D). In both roots and shoots, there were no similarities in expression patterns between GOI:WT and GOI:Null comparisons. For example, for TF *AP2-EREBP* genes in the roots, there was an upregulation of 23 DEGs in GOI:WT (with no downregulated DEGs), while in GOI:Null, there was an upregulation of only five and downregulation of four DEGs encoding this TF (Figure 5C). Furthermore, in the shoots, *WRKY* genes were most abundantly upregulated in GOI:WT (10 DEGs), but instead had 8 DEGs downregulated in GOI:Null (Figure 5D). Notably, there were a lot more DEGs encoding for TFs that were being upregulated in GOI:WT compared to GOI:Null in both roots and shoots (Figures 5C,D).

#### Metabolic Pathways Associated With DEGs

**FIGURE 6:**
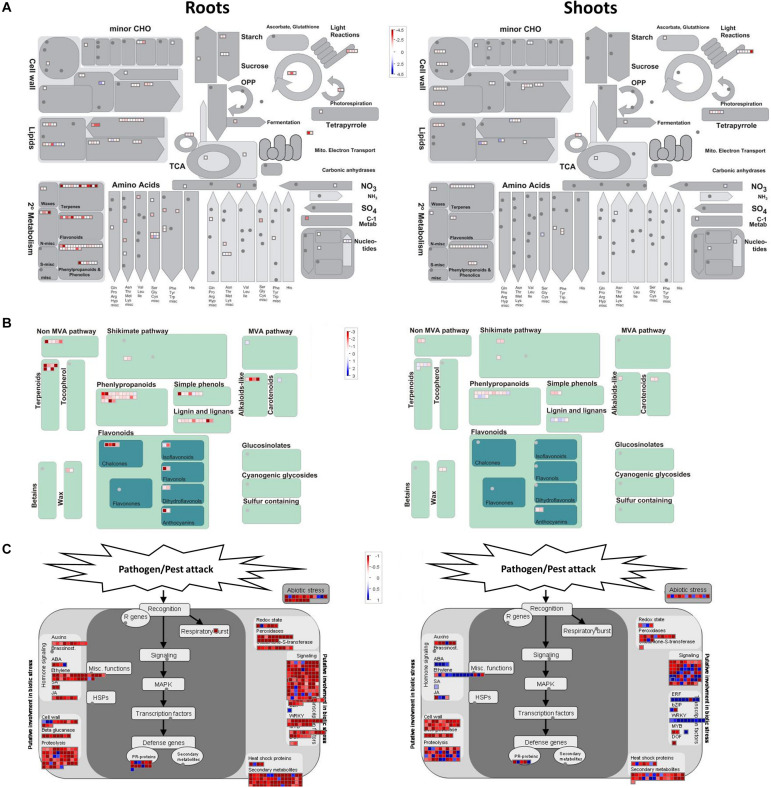
MapMan diagrams of differentially expressed genes between *OsAnt1:HvAlaAT* lines and wildtype for metabolism overview **(A)**, secondary metabolism **(B)**, and biotic stress response **(C)**. Color shades represent upregulation (red) or downregulation (blue) of genes.

Many of the identified DEGs encode enzymes involved in various metabolic pathways. We constructed MapMan figures for better visualizations of the specific processes within several categories that showed strong significance in GO enrichment. On this basis, metabolism overview, secondary metabolism, and biotic stress are presented in Figures 6A–C, respectively.

This observation tool identified clusters of gene activities related to secondary metabolism, most of which were upregulated in roots, particularly those involved in the non-Mevalonate (MVA) pathway, and in the production of flavonoids, phenylpropanoids, terpene, and terpenoids (Figure 6B). This is supported by the upregulated GO terms, which, for instance, showed that genes involved in terpene production *via* the non-MVA pathway were significantly upregulated (two terpene synthase genes, LOC_Os02g36140.4 and LOC_Os03g24690.1; Supplementary Table S5).

The MapMan representation of pathogen/pest attack (Figure 6C) also indicated many hormonal signaling pathways that were significantly upregulated in both roots and shoots, including ethylene production. Indeed, several genes involved in ethylene production, such as ethylene-responsive transcription factors (LOC_Os04g46220.1 and LOC_Os02g43790.1) and cystathionine gamma-synthase (LOC_Os10g25950.1 and LOC_Os10g37340.1) were upregulated in the transgenic rice lines (Figure 6B and Supplementary Table S5; Hacham et al., 2002; Avraham et al., 2005; Kim et al., 2006). However, these genes were also highlighted in the comparison between GOI and null (downregulated) or null and wildtype (upregulated) (Supplementary Figure S13). It is also interesting to note that clusters of gene activities related to cell wall synthesis were upregulated both in root and shoot (Figure 6C and Supplementary Figure S13).

## Discussion

### HvAlaAT Confers Advantageous Biomass Production and N Acquisition

The current study provided further evidence that in rice, the overexpression of *OsAnt1:HvAlaAT* can increase shoot biomass and grain production (Figure 1). This outcome is in agreement with previous studies (Shrawat et al., 2008; Beatty et al., 2013; Selvaraj et al., 2017), and demonstrated the ability of the transgene to confer a growth response in both controlled and non-controlled environments with a supply of N fertilizer (Figure 1). There was no N response in biomass in the growth chamber experiment (Figure 1A). This is probably due to the hydroponic system (i.e., 700 L capacity), which enables a steady N supply even at low concentration. When maize plants were grown at 0.5 and 2.5 mM N in the same system, no biomass difference was observed through the lifecycle (Garnett et al., 2013). In parallel studies, selected *OsAnt1:HvAlaAT* transgenic barley and wheat lines showed increased shoot biomass and seed production compared to the control plants (Figure 2). Across all of our studies, root biomass was unaffected in the transgenic rice lines, regardless of N treatment. This contradicts previous reports as *OsAnt1:HvAlaAT* rice lines produced higher root biomass than controls when the plants were grown in NO_3_^–^ (but not in NH_4_^+^; Shrawat et al., 2008), whereas Beatty et al. (2013) showed that the transgenic rice lines had increased shoot and, to some extent, root biomass as compared to controls at a range of NH_4_^+^ concentrations. In an African rice (NERICA) background, the *OsAnt1:HvAlaAT* transgenic lines showed no or limited increase in biomass of 43-day-old plants under low N condition (Selvaraj et al., 2017). These results suggest that biomass increase of the transgenic lines during the vegetative growth stage may be moderate at a non-significant level. Still, it is accumulative throughout the growth stages, resulting in a significant increase in biomass and seed yield. The physiological characterizations also provided a similar trend as we discuss next. Further testing of plant growth in different N regimes and growth systems over the lifecycle may present a clearer picture of the N response phenotype, including root architecture.

**FIGURE 7:**
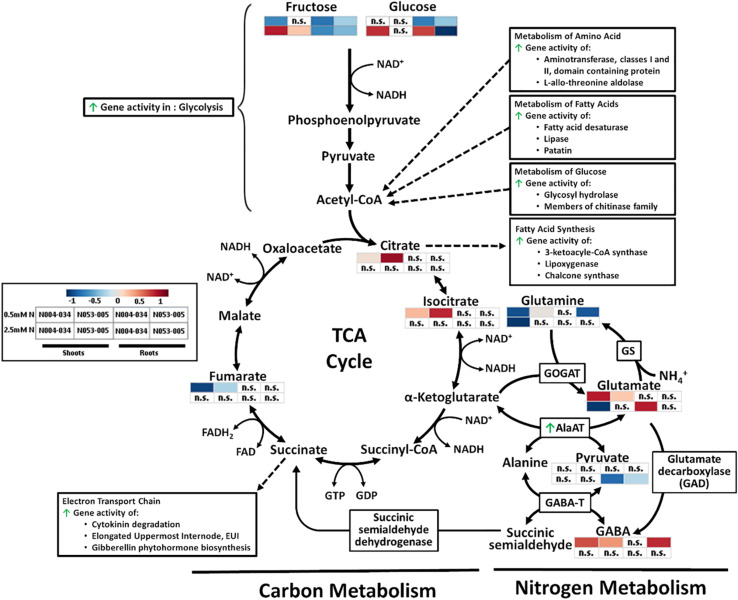
Simplified depiction of the influence of the *OsAnt1:HvAlaAT* transgene on the carbon and nitrogen metabolism pathway, consisting of glycolysis, TCA cycle, and GABA shunt. Increased gene activities are indicated with green arrows. Heatmaps of metabolites of the shoot and root where both transgenic lines show significant differences to WT in low N (0.5 mM N) and high N (2.5 mM N) are presented. Metabolite levels were processed by median normalization, log-transformed, and auto-scaling with MetaboAnalyst (Chong et al., 2019). Processes associated with certain metabolites were also presented (Acetyl-CoA, Citrate, and Succinate), with dotted arrows linking the aforementioned metabolites to represent either an end product (arrow direction toward it) or a substrate/catalyst (arrow direction away from it).

We observed no difference in NO_3_^–^ and NH_4_^+^ influx in *OsAnt1:HvAlaAT* lines (Figure 3), whereas Good et al. (2007) showed increased NO_3_^–^ HATS activity in canola lines overexpressing *HvAlaAT*. The contrasting findings may be due to the differences in growth condition and developmental stage when uptake was measured, or due to differences between species altogether. Although the N uptake step was not statistically significant, the rice transgenic lines might have some advantages in N acquisition, resulting in more N accumulation (e.g., N053-005) and increased biomass across the lifecycle (Figure 1 and Supplementary Figure S5). The increased glutamate and decreased glutamine levels in the shoots of low N plants (Figure 4 and Supplementary Table S3A) may be the results of disturbed N assimilation triggered by increased AlaAT activity. However, there was no evidence of a significant change of glutamine synthetase and glutamate synthase (GS-GOGAT) activity based on the DEGs analysis (Figure 7). Future studies involving N tracer isotopes could also help in determining the fate of N within different organs in transgenic lines.

### Which Is the Right Control, Wildtype vs. Null?

It is clear that the DEGs in the GOI:WT and GOI:Null comparisons display very differing patterns, agreeing with a large number of DEGs obtained in the Null:WT comparison (Figure 5A). This is unexpected, as null and wildtype should be genetically identical, and the DEGs between these two genotypes should be few in theory. However, our results suggest that wildtype and null differ to a degree at the transcriptional and metabolites level (Figures 4, 5). Interestingly, the null plants of GP4 barley transgenic lines also showed singnificantly lower grain yield compared to wildtype and the transgenic line (Figure 2B). Null segregants are often considered as the right controls as those lines also undergo the transformation process that includes *Agrobacterium*-mediated or particle bombardment transformation, cell culture, and hormone-induced organogenesis. Interestingly, it has been shown that *Agrobacterium*-mediated rice transgenic plants carried more unintended genomic mutations compared to particle bombardment or electroporation methods (Labra et al., 2001; Wilson et al., 2006). More recently, Fan et al. (2020b) reported the tissue culture process possibly causes epigenetic alterations in the rice genome. However, the stability of the epigenetic alterations may differ depending on the genetic background and may potentially be suppressed after some generations (Fan et al., 2020b). Those genomic alterations are heritable and independently segregate from GOI, which means it is possible that null segregants and GOI lines could carry different genomic profiles apart from the GOI. In this case, wildtype can be a better control as it has a less “noisy” genomic background. Our transgenic rice and barley lines were produced by *Agrobacterium*-mediated transformation, and that might explain the discrepancy between the nulls and wildtype. To isolate genetically cleaner nulls, one could backcross GOI with wildtype a couple of times before screening for nulls. In this way, pleiotropic effects from un-expected genomic alterations could be minimized. Alternatively, one could isolate a few null segregants from each transgenic event at T2, or T3 stage and characterize them individually along with wildtype.

### Overexpression of *AlaAT* Did Not Affect Alanine Levels

There were no differences in alanine levels across all genotypes and N treatments, despite the abundance in *HvAlaAT* gene expression in *OsAnt1:HvAlaAT* lines (Supplementary Figure S10A). Our growth experiments with these rice lines showed increased enzymatic activity of total AlaAT in both roots and shoots grown in a mixed N source [NH_4_NO_3_ and Ca(NO_3_)_2_] (Supplementary Figure S9). This is consistent with other reports on similar rice lines (Shrawat et al., 2008; Beatty et al., 2013). We also confirmed the presence of the expressed HvAlaAT protein in the rice lines (Supplementary Figure S8B; Skinner et al., 2012). The unchanged alanine and glutamate levels in the roots of the transgenic lines (Supplementary Tables S3A,B, S4A,B) could potentially be due to rapid assimilation in the roots, and/or translocation to the shoots for protein synthesis. The increased glutamate levels in the shoots of low N plants (Supplementary Table S3A) suggests that the latter might have occurred.

Increased GABA and glutamate levels in shoots of low N plants (Supplementary Table S3A) could be evidence of increased AlaAT activity; the GABA shunt pathway, which produces GABA, involves the conversion of glutamate to GABA as catalyzed by glutamate decarboxylase (Figure 7; Steinhauser et al., 2012). Upregulation of AlaAT activity may have increased the conversion of α-Ketoglutarate to glutamate, which could have subsequently increased GABA production (Supplementary Table S3A).

### Emerging Clues for the Mechanisms of *OsAnt1:HvAlaAT* Conferring NUE Phenotype

#### Up-Regulation of Carbohydrate Metabolic Pathways

##### Changes in glycolysis

Overexpression of *OsAnt1:HvAlaAT* may have increased the conversion of pyruvate to alanine (at least when grown under low N), which in turn caused negative feedback to increase glycolysis activity (Figure 7). Evidence of increased glycolysis activity is, firstly, the significant reduction of glucose and fructose in roots under low N (Supplementary Table S3B). Glucose is a direct precursor metabolite of glycolysis, while fructose may be funneled into the glycolysis pathway by conversion to dihydroxyacetone phosphate (*via* fructokinase and F-1-P aldolase), an intermediate in glycolysis (Berg et al., 2012). Secondly, there are upregulated expression levels of genes involved in glucose metabolisms, such as glycosyl hydrolases and members of the chitinase family (Supplementary Tables S5, S6). On a broader scope, some glycolysis-associated genes were upregulated. Those include lactate dehydrogenase (LDH; catalyzes the conversion of L-lactate to pyruvate, the last step in anaerobic glycolysis), and fructose-bisphospate aldolase isozyme (key enzyme in the fourth step of glycolysis). While LDH is not part of the main glycolysis cycle, it catalyzes the conversion of pyruvate (the last product in anaerobic glycolysis) to lactate (Christopher and Good, 1996). The lack of increase in pyruvate in the transgenic line (Figure 4 and Supplementary Tables S3, S4) could be due to increased activity of LDH in converting pyruvate to lactate. Future studies on measuring lactate and the activity of rate-limiting glycolytic enzymes such as hexokinases, phosphofructokinase, and pyruvate kinases can help us confirm if the overexpression of *OsAnt1:HvAlaAT* had affected glycolysis.

##### Changes in the TCA cycle

Evidence of increased TCA cycle activity is the altered level of metabolites such as fumarate, citrate and isocitrate in shoots under low N (Supplementary Table S3A and Figures 4, 7). In addition, numerous genes for enzymes that use substrates from the TCA cycle were significantly upregulated (Supplementary Tables S5, S6). The enzymes include those which drive amino acid metabolism (aminotransferase, and L-allo-threonine aldolase; Jander et al., 2004), glucose metabolism (glycosyl hydrolase and members of chitinase family; Sinnott, 1990; Ashhurst, 2001), and fatty acid metabolism and synthesis (fatty acid desaturase, lipase, patatin, 3-ketoacyl-CoA synthase, lipoxygenase and chalcone synthase; Post-Beittenmiller et al., 1992; Rustérucci et al., 1999). All of these pathways produce acetyl-CoA that feeds into the TCA cycle (Figure 7; Kim et al., 1989). Under high N, pyruvate levels were elevated in the roots of *OsAnt1:HvAlaAT* lines (Supplementary Table S4B), implying that lesser pyruvate was utilized under high N, which may indicate lowered TCA cycle activity, leading to lesser ATP production (hence, absence of growth advantage; Berg et al., 2012).

There were increases in the expression of genes involved in the electron transport chain, a process downstream of the TCA cycle (Supplementary Tables S5, S6 and Figure 7). Many of these genes have a role in regulating plant growth and development, such as those involved in cytokinin degradation (cytokinin is linked with controlling plant growth such as leaf expansion and photosynthesis; Peleg and Blumwald, 2011), Elongated Uppermost Internode (EUI; important components of the gibberellic acid biosynthetic and signal transduction pathways regulating plant height; Zhu et al., 2006), and gibberellin (GA) phytohormone biosynthesis (Supplementary Tables S5, S6). Expressing *OsAnt1:HvAlaAT* may have perturbed pyruvate levels, affecting the level of TCA cycle activity. This could result in more energy being produced to promote N uptake and usage, and subsequently, plant growth and development (Kim et al., 2006; Murchie et al., 2009).

#### Up-Regulation of Ethylene Production

As shown earlier, several genes involved in ethylene production were upregulated in the transgenic rice lines compared to wildtype (Figure 6C and Supplementary Table S5). Ethylene is a key plant hormone formed in response to biotic and abiotic stresses. For example, ethylene can regulate the growth of roots to cope with hypoxia during flooding (Geisler-Lee et al., 2010), and promotes growth-related characteristics, such as stem elongation, seed germination, and senescence. ATP has a key involvement in the biosynthesis of ethylene (Wang et al., 2002). It is tempting to suggest that higher ethylene biosynthesis activity (Figure 6B), which may be further induced by the higher availability of ATP due to an upregulated TCA cycle, resulted in higher biomass production (Figure 1). As endogenous *AlaAT* is induced by hypoxia (Good and Crosby, 1989), the over-expression of *HvAlaAT* could be registered as a sign of hypoxic stress, causing the plants to increase ethylene biosynthesis, which subsequently improves the uptake efficiency of roots in obtaining N (Geisler-Lee et al., 2010; Lemaire et al., 2013; Khan et al., 2015). On the other hand, Null:WT comparison revealed significant numbers of hormonal and stress-related genes, suggesting that the transformation process might have triggered the unique expression profiles as discussed earlier (Supplementary Figure S13C). Nevertheless, it is noteworthy that some other hormone-related genes are upregulated in the transgenic line compared to both wildtype and the null (Figure 5B and Supplementary Table S9). The genes include gibberellin (GA) 2-beta-dioxygenase 7 (*GA2OX7*, LOC_Os7g01340), GA receptor (*GID1*, LOC_Os06g11135, LOC_Os07g44900), auxin-induced protein (LOC_Os8g44750). Orthologs of *OsGA2OX7* in maize (*ZmGA2AOX12*) and Arabidopsis (*AtGA2AOX7*) are involved in stem elongation (Schomburg et al., 2003) and stress response (Lange and Lange, 2015; Hoopes et al., 2019). GID1 is an essential component in the GA signaling and induced by biotic and abiotic stresses in rice (Ueguchi-Tanaka et al., 2005; Du et al., 2015; Chen et al., 2018). Taken together, the hormonal responses to *HvAlaAT* gene expression might be a part of the improved NUE phenotype, and it will therefore be interesting to measure multiple phytohormone levels in *OsAnt1:HvAlaAT* lines by a hormonomics approach (Šimura et al., 2018).

#### Other Factors Which Could Confer NUE Phenotype

Differential gene expression and MapMan analysis also showed that gene activities related to secondary metabolism and the production of secondary metabolites were upregulated (Figure 6 and Supplementary Table S5). Secondary metabolites are important for producing photosynthetic compounds and carbohydrate metabolism (Gould et al., 2000; Close and McArthur, 2002; Beatty et al., 2013; Sandquist and Ehleringer, 2014). Indeed, the upregulation of genes involved in the synthesis of chloroplast and chlorophyll was observed (Supplementary Tables S5, S6). It will thus be beneficial to measure the photosynthetic rate in *OsAnt1:HvAlaAT* lines in the future.

One notable observation from the metabolic data is the decrease in arabinose in both shoots and roots of low N plants (Supplementary Tables S3A,B). Hydroxyproline-rich glycoproteins (HRGP) contain numerous arabinose side chains and play a structural role in strengthening the cell wall and often are expressed in response to pathogen attack (Showalter, 1993). Lowered arabinose levels could indicate higher synthesis of HRGP, which may lead to the observed higher biomass production (Showalter et al., 2016; Johnson et al., 2017) in *OsAnt1:HvAlaAT* lines. The upregulation of gene activities involved in cell wall synthesis and response to pathogen attack (biotic stress) as indicated by MapMan (Figure 6C) may also suggest up-regulation of HRGP synthesis.

Another interesting observation from the metabolic data is the higher glycine levels in the shoots of low N plants (Supplementary Table S3A). Glycine is a metabolite of photorespiration, but also known to be involved in cellular macromolecule protection and ROS detoxification (Giri, 2011), and accumulates in response to environmental stresses such as drought and salinity (Ashraf and Foolad, 2007). This suggests that glycine can confer positive traits such as enzyme and membrane integrity under environmental stresses, and its higher accumulation in *OsAnt1:HvAlaAT* lines may have thus promoted overall higher biomass production.

## Conclusion

We investigated the effect of expressing *HvAlaAT* in rice, barley and wheat. The transgenic plants showed some advantages in growth and seed production. Comprehensive analyses were conducted using rice lines to unravel the molecular characteristics that could lead to the growth advantage. The altered expression of *AlaAT* has resulted in significant changes within the carbohydrate metabolism pathway that includes glycolysis and the TCA cycle. This may have driven increased energy production to promote improved N assimilation and utilization, leading to higher biomass production. The current study is an interesting example of crop improvement, altering downstream of N assimilation by “pulling” N demand rather than “pushing” N into plants. The small difference may not be significant at a single time point, however, the change could be effective over the growth period, resulting in a significant increase in biomass and yield as we have shown. Perhaps, constant phenotyping or multiple points of harvest across the lifecycle may unveil further evidence for the mechanism of the NUE technology.

## Data Availability Statement

The datasets presented in this study can be found in online repositories. The names of the repository/repositories and accession number(s) can be found below: NCBI-SRA, PRJNA687265.

## Author Contributions

JT, ZL, JK, BK, and MO conceived and designed the experiments. JT, NS, RS, NM, SW, CM, and ZL grew plants and conducted the experiments. WS, YL, and JK carried out the metabolic and protein analyses. NS and UB performed the bioinformatics analyses. JT and MO wrote the manuscript, and JK and SH critically read it. All authors contributed to the article and approved the submitted version.

## Conflict of Interest

JK was a named inventor on United States Patents Nitrogen-Efficient Monocot Plants US 8642840 and 8288611, and Arcadia Biosciences has exclusively licensed these patents and others concerning the NUE technology described. JK, WS, YL, and ZL are employees of Arcadia Biosciences, Inc. and as such Arcadia partially funded the work described. The remaining authors declare that the research was conducted in the absence of any commercial or financial relationships that could be construed as a potential conflict of interest.

## Footnotes

1 http://www.bioinformatics.babraham.ac.uk/projects/fastqc/

2 http://plntfdb.bio.uni-potsdam.de/v3.0/

## Supplementary Material

Supplementary Figure 1 Effects of low and adequate N treatment on root biomass of rice plants expressing *OsAnt1:HvAlaAT.*

Supplementary Figure 2 Effects of low and adequate N treatment on harvest index of rice plants expressing *OsAnt1:HvAlaAT.*

Supplementary Figure 3 Grain yield of rice plants (*Oryza sativa*, cv. Taipei) expressing *OsAnt1:HvAlaAT* grown in the field.

Supplementary Figure 4 Effects of low and adequate N treatment on nitrate accumulation in shoots and roots of rice plants expressing *OsAnt1:HvAlaAT.*

Supplementary Figure 5 Effects of low and adequate N treatment on ammonium accumulation in shoots and roots of rice plants expressing *OsAnt1:HvAlaAT*.

Supplementary Figure 6 Effects of low and adequate N treatment on total N accumulation in rice plants expressing *OsAnt1:HvAlaAT*.

Supplementary Figure 7 Effects of low and adequate N treatment on nitrate and ammonium uptake in rice plants (*Oryza sativa*, cv. Taipei) expressing *OsAnt1:HvAlaAT* via high- and low-affinity transport systems (HATS and LATS).

Supplementary Figure 8 Barley *AlaAT* (*HvAlaAT*) copy number and expression of HvAlaAT protein in transgenic rice plants.

Supplementary Figure 9 AlaAT enzymatic activity in rice plants expressing *OsAnt1:HvAlaAT*.

Supplementary Figure 10 Quantitative RT-PCR analysis of *OsAnt1:AlaAT* lines and wildtype.

Supplementary Figure 11 GO term assignment of differentially expressed transcripts between *OsAnt1:HvAlaAT* lines (GOI) and wildtype (WT) controls in different categories of cellular component, molecular function and biological processes for roots and shoots.

Supplementary Figure 12 GO enrichment of differentially expressed genes *OsAnt1:HvAlaAT* lines and wildtype.

Supplementary Figure 13 MapMan diagrams of differentially expressed genes between *OsAnt1:HvAlaAT* lines and Nulls and between Nulls and wildtype for biotic stress response.

Supplementary Table 1 Summary of every experiment performed with each crop species expressing *OsAnt1:HvAlaAT*.

Supplementary Table 2 Primer sequences and identifiers for the rice genes analyzed by Quantitative Reverse Transcript Polymerase Chain Reaction (QRT-PCR).

Supplementary Table 3 Comparison of metabolites (μg g^–1^ DW) in shoots and roots between *OsAnt1:HvAlaAT* lines and wildtype and between *OsAnt1:HvAlaAT* lines and nulls grown in low nitrogen (0.5 mM N).

Supplementary Table 4 Comparison of metabolites (μg g^–1^ DW) in shoots and roots between *OsAnt1:HvAlaAT* lines and wildtype and between *OsAnt1:HvAlaAT* lines and nulls grown in high nitrogen (2.5 mM N).

Supplementary Table 5 Differentially expressed transcripts for comparison between *OsAnt1:HvAlaAT* lines (GOI) and wildtype (WT) controls in roots.

Supplementary Table 6 Differentially expressed transcripts for comparison between *OsAnt1:HvAlaAT* lines (GOI) and wildtype (WT) controls in shoots.

Supplementary Table 7 Differentially expressed transcripts for comparison between *OsAnt1:HvAlaAT* lines (GOI) and Null controls in roots.

Supplementary Table 8 Differentially expressed transcripts for comparison between *OsAnt1:HvAlaAT* lines (GOI) and Null controls in shoots.

Supplementary Table 9 Overlapping differentially expressed transcripts between *OsAnt1:HvAlaAT* lines (GOI): wildtype (WT) controls and GOI:Null controls in roots and shoots.

Supplementary Table 10 Gene Ontology (GO) term assignment of the overlapped differentially expressed transcripts between GOI:WT and GOI:Null.

Supplementary Method AlaAT protein expression and enzyme activity.

## References

[B1] Ashhurst D. E. (2001). Chitin and chitinases. *Cell Biochem. Funct.* 19 228–228. 10.1002/cbf.916

[B2] Ashraf M. Foolad M. R. (2007). Roles of glycine betaine and proline in improving plant abiotic stress resistance. *Environ. Exp. Bot.* 59 206–216. 10.1016/j.envexpbot.2005.12.006

[B3] Avice J.-C. Etienne P. (2014). Leaf senescence and nitrogen remobilization efficiency in oilseed rape (Brassica napus L.). *J. Exp. Bot.* 65 3813–3824. 10.1093/jxb/eru177 24790115

[B4] Avraham T. Badani H. Galili S. Amir R. (2005). Enhanced levels of methionine and cysteine in transgenic alfalfa (Medicago sativa L.) plants over-expressing the Arabidopsis cystathionine γ-synthase gene. *Plant Biotechnol. J.* 3 71–79. 10.1111/j.1467-7652.2004.00102.x 17168900

[B5] Beatty P. H. Carroll R. T. Shrawat A. K. Guevara D. Good A. G. (2013). Physiological analysis of nitrogen-efficient rice overexpressing alanine aminotransferase under different N regimes. *Bot. Botanique* 91 866–883. 10.1139/cjb-2013-0171

[B6] Berg J. M. Tymoczko J. L. Stryer L. (2012). *Biochemistry.* Basingstoke: W.H. Freeman.

[B7] Borrell A. Garside A. Fukai S. Reid D. (1998). Season, nitrogen rate, and plant type affect nitrogen uptake and nitrogen use efficiency in rice. *Aust. J. Agric. Res.* 49 829–843. 10.1071/A97057

[B8] Cataldo D. A. Haroon M. Schrader L. E. Youngs V. L. (1975). Rapid colorimetric determination of nitrate in plant tissue by nitration of salicylic acid. *Commun. Soil Sci. Plant Anal.* 6 71–80. 10.1080/00103627509366547

[B9] Chen L. Cao T. Zhang J. Lou Y. (2018). Overexpression of *OsGID1* enhances the resistance of rice to the brown planthopper *Nilaparvata lugens*. *Int. J. Mol. Sci.* 19:2744. 10.3390/ijms19092744 30217023 PMC6164479

[B10] Chong J. Wishart D. S. Xia J. (2019). Using MetaboAnalyst 4.0 for Comprehensive and Integrative Metabolomics Data Analysis. *Curr. Protoc. Bioinfor.* 68:e86. 10.1002/cpbi.86 31756036

[B11] Christopher M. E. Good A. G. (1996). Characterization of hypoxically inducible lactate dehydrogenase in maize. *Plant Physiol.* 112, 1015–1022. 10.1104/pp.112.3.1015 12226430 PMC158028

[B12] Ciampitti I. A. Vyn T. J. (2012). Physiological perspectives of changes over time in maize yield dependency on nitrogen uptake and associated nitrogen efficiencies: A review. *Field Crops Res.* 133 48–67. 10.1016/j.fcr.2012.03.008

[B13] Close D. C. McArthur C. (2002). Rethinking the role of many plant phenolics–protection from photodamage not herbivores? *Oikos* 99 166–172. 10.1034/j.1600-0706.2002.990117.x 11841302

[B14] Crawford N. M. Glass A. D. M. (1998). Molecular and physiological aspects of nitrate uptake in plants. *Trends Plant Sci.* 3 389–395. 10.1016/S1360-1385(98)01311-9

[B15] DoVale J. Delima R. Fritsche-Neto R. (2012). “Breeding for Nitrogen Use Efficiency,” in *Plant Breeding for Abiotic Stress Tolerance*, eds Fritsche-Neto R. Borém A. (Berlin: Springer), 53–65.

[B16] Druka A. Kudrna D. Kannangara C. G. Von Wettstein D. Kleinhofs A. (2002). Physical and genetic mapping of barley (Hordeum vulgare) germin-like cDNAs. *Proc. Natl. Acad. Sci. U S A.* 99 850–855. 10.1073/pnas.022627999 11792854 PMC117394

[B17] Du H. Chang Y. Huang F. Xiong L. (2015). GID1 modulates stomatal response and submergence tolerance involving abscisic acid and gibberellic acid signaling in rice. *J. Integr. Plant Biol.* 57 954–968. 10.1111/jipb.12313 25418692

[B18] Fan T. Yang W. Zeng X. Xu X. L. Xu Y. L. Fan X. R. (2020a). A Rice Autophagy GeneOsATG8bIs Involved in Nitrogen Remobilization and Control of Grain Quality. *Front. Plant Sci.* 11:588. 10.3389/fpls.2020.00588 32582228 PMC7287119

[B19] Fan X. Chen J. Wu Y. Teo C. Xu G. Fan X. (2020b). Genetic and global epigenetic modification, which determines the phenotype of transgenic rice? *Int. J. Mol. Sci.* 21:1819. 10.3390/ijms21051819 32155767 PMC7084647

[B20] Fan X. Tang Z. Tan Y. Zhang Y. Luo B. Yang M. (2016). Overexpression of a pH-sensitive nitrate transporter in rice increases crop yields. *Proc. Natl. Acad. Sci. U S A.* 113 7118–7123. 10.1073/pnas.1525184113 27274069 PMC4932942

[B21] Garnett T. Conn V. Plett D. Conn S. Zanghellini J. Mackenzie N. (2013). The response of the maize nitrate transport system to nitrogen demand and supply across the lifecycle. *N. Phytol.* 198 82–94. 10.1111/nph.12166 23398565

[B22] Geisler-Lee J. Caldwell C. Gallie D. R. (2010). Expression of the ethylene biosynthetic machinery in maize roots is regulated in response to hypoxia. *J. Exp. Bot.* 61 857–871. 10.1093/jxb/erp362 20008461 PMC2814119

[B23] Giri J. (2011). Glycinebetaine and abiotic stress tolerance in plants. *Plant Signal. Behav.* 6 1746–1751. 10.4161/psb.6.11.17801 22057338 PMC3329348

[B24] Good A. G. Crosby W. L. (1989). Anaerobic induction of alanine aminotransferase in barley root tissue. *Plant Physiol.* 90 1305–1309. 10.1104/pp.90.4.1305 16666927 PMC1061887

[B25] Good A. G. Muench D. G. (1993). Long-Term Anaerobic Metabolism in Root Tissue (Metabolic Products of Pyruvate Metabolism). *Plant Physiol.* 101 1163–1168. 10.1104/pp.101.4.1163 12231768 PMC160634

[B26] Good A. G. Johnson S. J. De Pauw M. Carroll R. T. Savidov N. Vidmar J. (2007). Engineering nitrogen use efficiency with alanine aminotransferase. *Can. J. Bot.* 85 252–262. 10.1139/B07-019

[B27] Gould K. S. Markham K. R. Smith R. H. Goris J. J. (2000). Functional role of anthocyanins in the leaves of *Quintinia serrata* A. *Cunn. J. Exp. Bot.* 51 1107–1115. 10.1093/jexbot/51.347.1107 10948238

[B28] Hacham Y. Avraham T. Amir R. (2002). The N-terminal region of Arabidopsis cystathionine γ-synthase plays an important regulatory role in methionine metabolism. *Plant Physiol.* 128 454–462. 10.1104/pp.010819 11842149 PMC148908

[B29] Hawkesford M. J. (2014). Reducing the reliance on nitrogen fertilizer for wheat production. *J. Cereal Sci.* 59 276–283. 10.1016/j.jcs.2013.12.001 24882935 PMC4026125

[B30] Hirai M. Y. Yano M. Goodenowe D. B. Kanaya S. Kimura T. Awazuhara M. (2004). Integration of transcriptomics and metabolomics for understanding of global responses to nutritional stresses in Arabidopsis thaliana. *Proc. Natl. Acad. Sci. U S A.* 101 10205–10210. 10.1073/pnas.0403218101 15199185 PMC454188

[B31] Hirel B. Le Gouis J. Ney B. Gallais A. (2007). The challenge of improving nitrogen use efficiency in crop plants: towards a more central role for genetic variability and quantitative genetics within integrated approaches. *J. Exp. Bot.* 58 2369–2387. 10.1093/jxb/erm097 17556767

[B32] Hoopes G. M. Hamilton J. P. Wood J. C. Esteban E. Pasha A. Vaillancourt B. (2019). An updated gene atlas for maize reveals organ-specific and stress-induced genes. *Plant J.* 97 1154–1167. 10.1111/tpj.14184 30537259 PMC6850026

[B33] Jaiswal P. Ni J. Yap I. Ware D. Spooner W. Youens-Clark K. (2006). Gramene: a bird’s eye view of cereal genomes. *Nucl. Acids Res.* 34 D717–D723. 10.1093/nar/gkj154 16381966 PMC1347516

[B34] Jander G. Norris S. R. Joshi V. Fraga M. Rugg A. Yu S. (2004). Application of a high-throughput HPLC-MS/MS assay to Arabidopsis mutant screening; evidence that threonine aldolase plays a role in seed nutritional quality. *Plant J.* 39 465–475. 10.1111/j.1365-313X.2004.02140.x 15255874

[B35] Johnson C. Stout P. Broyer T. C. Carlton A. B. (1957). Comparative chlorine requirements of different plant species. *Plant Soil* 8 337–353. 10.1007/BF01666323

[B36] Johnson K. L. Cassin A. M. Lonsdale A. Bacic A. Doblin M. S. Schultz C. J. (2017). A motif and amino acid bias bioinformatics pipeline to identify hydroxyproline-rich glycoproteins. *Plant Physiol.* 174, 886–903. 10.1104/pp.17.00294 28446635 PMC5462032

[B37] Khan M. I. R. Trivellini A. Fatma M. Masood A. Francini A. Iqbal N. (2015). Role of ethylene in responses of plants to nitrogen availability. *Front. Plant Sci.* 6:927. 10.3389/fpls.2015.00927 26579172 PMC4626634

[B38] Kim K. H. López-Casillas F. Bai D. H. Luo X. Pape M. E. (1989). Role of reversible phosphorylation of acetyl-CoA carboxylase in long-chain fatty acid synthesis. *FASEB J.* 3 2250–2256. 10.1096/fasebj.3.11.2570725 2570725

[B39] Kim S.-Y. Sivaguru M. Stacey G. (2006). Extracellular ATP in plants. Visualization, localization, and analysis of physiological significance in growth and signaling. *Plant Physiol.* 142 984–992. 10.1104/pp.106.085670 16963521 PMC1630726

[B40] Kovalchuk N. Smith J. Pallotta M. Singh R. Ismagul A. Eliby S. (2009). Characterization of the wheat endosperm transfer cell-specific protein TaPR60. *Plant Mol. Biol.* 71 81–98. 10.1007/s11103-009-9510-1 19513805

[B41] Kronzucker H. J. Siddiqi M. Y. Glass A. D. (1995). Kinetics of NO_3_^–^influx in spruce. *Plant Physiol.* 109 319–326. 10.1104/pp.109.1.319 12228598 PMC157591

[B42] Kumar A. Kaiser B. N. Siddiqi M. Y. Glass A. D. M. (2006). Functional characterisation of OsAMT1.1 overexpression lines of rice, Oryza sativa. *Funct. Plant Biol.* 33 339–346. 10.1071/fp05268 32689240

[B43] Kurai T. Wakayama M. Abiko T. Yanagisawa S. Aoki N. Ohsugi R. (2011). Introduction of the ZmDof1 gene into rice enhances carbon and nitrogen assimilation under low-nitrogen conditions. *Plant Biotechnol. J.* 9 826–837. 10.1111/j.1467-7652.2011.00592.x 21624033

[B44] Labra M. Savini C. Bracale M. Pelucchi N. Colombo L. Bardini M. (2001). Genomic changes in transgenic rice (Oryza sativa L.) plants produced by infecting calli with *Agrobacterium tumefaciens*. *Plant Cell Rep.* 20 325–330. 10.1007/s002990100329

[B45] Ladha J. K. Tirol-Padre A. Reddy C. K. Cassman K. G. Verma S. Powlson D. S. (2016). Global nitrogen budgets in cereals: A 50-year assessment for maize, rice, and wheat production systems. *Sci. Rep.* 6:19355. 10.1038/srep19355 26778035 PMC4726071

[B46] Lange M. J. P. Lange T. (2015). Touch-induced changes in Arabidopsis morphology dependent on gibberellin breakdown. *Nat. Plants* 1:14025. 10.1038/nplants.2014.25 27246879

[B47] Langmead B. Salzberg S. L. (2012). Fast gapped-read alignment with Bowtie 2. *Nat. Methods* 9 357–359. 10.1038/nmeth.1923 22388286 PMC3322381

[B48] Lassaletta L. Billen G. Grizzetti B. Anglade J. Garnier J. (2014). 50 year trends in nitrogen use efficiency of world cropping systems: the relationship between yield and nitrogen input to cropland. *Environ. Res. Lett.* 9:105011 10.1088/1748-9326/9/10/105011

[B49] Le Gouis J. Béghin D. Heumez E. Pluchard P. (2000). Genetic differences for nitrogen uptake and nitrogen utilisation efficiencies in winter wheat. *Eur. J. Agron.* 12 163–173. 10.1016/S1161-0301(00)00045-9

[B50] Lemaire L. Deleu C. Le Deunff E. (2013). Modulation of ethylene biosynthesis by ACC and AIB reveals a structural and functional relationship between the K^15^NO_3_ uptake rate and root absorbing surfaces. *J. Exp. Bot.* 64 2725–2737. 10.1093/jxb/ert124 23811694

[B51] Li H. Handsaker B. Wysoker A. Fennell T. Ruan J. Homer N. (2009). The Sequence Alignment/Map format and SAMtools. *Bioinformatics* 25 2078–2079. 10.1093/bioinformatics/btp352 19505943 PMC2723002

[B52] Maere S. Heymans K. Kuiper M. (2005). BiNGO: a Cytoscape plugin to assess overrepresentation of gene ontology categories in biological networks. *Bioinformatics* 21 3448–3449. 10.1093/bioinformatics/bti551 15972284

[B53] Matthews P. R. Wang M.-B. Waterhouse P. M. Thornton S. Fieg S. J. Gubler F. (2001). Marker gene elimination from transgenic barley, using co-transformation with adjacent ‘twin T-DNAs’ on a standard Agrobacterium transformation vector. *Mol. Breed.* 7 195–202. 10.1023/a:1011333321893

[B54] Mauch F. Reimmann C. Freydl E. Schaffrath U. Dudler R. (1998). Characterization of the rice pathogen-related protein Rir1a and regulation of the corresponding gene. *Plant Mol. Biol.* 38 577–586. 10.1023/A:1006041404436 9747803

[B55] McAllister C. H. Beatty P. H. Good A. G. (2012). Engineering nitrogen use efficient crop plants: the current status. *Plant Biotechnol. J.* 10 1011–1025. 10.1111/j.1467-7652.2012.00700.x 22607381

[B56] Miyashita Y. Dolferus R. Ismond K. P. Good A. G. (2007). Alanine aminotransferase catalyses the breakdown of alanine after hypoxia in *Arabidopsis thaliana*. *Plant J.* 49 1108–1121. 10.1111/j.1365-313X.2006.03023.x 17319845

[B57] Mosleth E. F. Wan Y. Lysenko A. Chope G. A. Penson S. P. Shewry P. R. (2015). A novel approach to identify genes that determine grain protein deviation in cereals. *Plant Biotechnol. J.* 13 625–635. 10.1111/pbi.12285 25400203

[B58] Muench D. G. Good A. G. (1994). Hypoxically inducible barley alanine aminotransferase: cDNA cloning and expression analysis. *Plant Mol. Biol.* 24 417–427. 10.1007/BF00024110 8123785

[B59] Murchie E. Pinto M. Horton P. (2009). Agriculture and the new challenges for photosynthesis research. *N. Phytol.* 181 532–552. 10.1111/j.1469-8137.2008.02705.x 19140947

[B60] Nauer E. M. Labanauskas C. K. Roistacher C. N. (1967). Effects of mix composition, fertilization, and pH on citrus grown in U.C.-Type potting mixtures under greenhouse conditions. *Hilgardia* 38 557–567. 10.3733/hilg.v38n15p557

[B61] Okamoto M. Vidmar J. J. Glass A. D. M. (2003). Regulation of *NRT1* and *NRT2* gene families of *Arabidopsis thaliana*: Responses to nitrate provision. *Plant Cell Physiol.* 44 304–317. 10.1093/pcp/pcg036 12668777

[B62] Ortiz-Monasterio J. I. Sayre K. D. Rajaram S. Mcmahon M. (1997). Genetic progress in wheat yield and nitrogen use efficiency under four nitrogen rates. *Crop Sci.* 37 898–904. 10.2135/cropsci1997.0011183X003700030033x

[B63] Passardi F. Longet D. Penel C. Dunand C. (2004). The class III peroxidase multigenic family in rice and its evolution in land plants. *Phytochemistry* 65 1879–1893. 10.1016/j.phytochem.2004.06.023 15279994

[B64] Peleg Z. Blumwald E. (2011). Hormone balance and abiotic stress tolerance in crop plants. *Curr. Opin. Plant Biol.* 14 290–295. 10.1016/j.pbi.2011.02.001 21377404

[B65] Peña P. A. Quach T. Sato S. Ge Z. Nersesian N. Dweikat I. M. (2017). Molecular and phenotypic characterization of transgenic wheat and sorghum events expressing the barley alanine aminotransferase. *Planta* 246 1097–1107. 10.1007/s00425-017-2753-1 28801748

[B66] Post-Beittenmiller D. Roughan G. Ohlrogge J. B. (1992). Regulation of Plant Fatty Acid Biosynthesis: Analysis of Acyl-Coenzyme A and Acyl-Acyl Carrier Protein Substrate Pools in Spinach and Pea Chloroplasts. *Plant Physiol.* 100 923–930. 10.1104/pp.100.2.923 16653077 PMC1075645

[B67] Raun W. R. Johnson G. V. (1999). Improving nitrogen use efficiency for cereal production. *Agron. J.* 91 357–363. 10.2134/agronj1999.00021962009100030001x

[B68] Roessner U. Wagner C. Kopka J. Trethewey R. N. Willmitzer L. (2000). Simultaneous analysis of metabolites in potato tuber by gas chromatography–mass spectrometry. *Plant J.* 23 131–142. 10.1046/j.1365-313x.2000.00774.x 10929108

[B69] Rustérucci C. Montillet J.-L. Agnel J.-P. Battesti C. Alonso B. Knoll A. (1999). Involvement of Lipoxygenase-dependent Production of Fatty Acid Hydroperoxides in the Development of the Hypersensitive Cell Death induced by Cryptogein on Tobacco Leaves. *J. Biol. Chem.* 274 36446–36455. 10.1074/jbc.274.51.36446 10593941

[B70] Sadras V. O. Lawson C. (2013). Nitrogen and water-use efficiency of Australian wheat varieties released between 1958 and 2007. *Eur. J. Agron.* 46 34–41. 10.1016/j.eja.2012.11.008

[B71] Sandquist D. Ehleringer J. (2014). “Photosynthesis: physiological and ecological considerations,” in *Plant Physiology and Development*, eds Taiz L. Zeiger E. Møller I. M. Murphy A. (Massachusetts: Sinauer Associates).

[B72] Schomburg F. M. Bizzell C. M. Lee D. J. Zeevaart J. A. D. Amasino R. M. (2003). Overexpression of a Novel Class of Gibberellin 2-Oxidases Decreases Gibberellin Levels and Creates Dwarf Plants. *Plant Cell* 15 151–163. 10.1105/tpc.005975 12509528 PMC143488

[B73] Selvaraj M. G. Valencia M. O. Ogawa S. Lu Y. Wu L. Downs C. (2017). Development and field performance of nitrogen use efficient rice lines for Africa. *Plant Biotechnol. J.* 15 775–787. 10.1111/pbi.12675 27889933 PMC5425388

[B74] Showalter A. M. (1993). Structure and function of plant cell wall proteins. *Plant Cell* 5 9–23. 10.1105/tpc.5.1.9 8439747 PMC160246

[B75] Showalter A. M. Keppler B. D. Liu X. Lichtenberg J. Welch L. R. (2016). Bioinformatic identification and analysis of hydroxyproline-rich glycoproteins in populus trichocarpa. *BMC Plant Biol.* 16:229. 10.1186/s12870-016-0912-3 27769192 PMC5073881

[B76] Shrawat A. K. Carroll R. T. Depauw M. Taylor G. J. Good A. G. (2008). Genetic engineering of improved nitrogen use efficiency in rice by the tissue-specific expression of alanine aminotransferase. *Plant Biotechnol. J.* 6 722–732. 10.1111/j.1467-7652.2008.00351.x 18510577

[B77] Siddiqi M. Y. Glass A. D. M. Ruth T. J. Rufty T. W. Jr. (1990). Studies of the uptake of nitrate in barley I. Kinetics of ^13^NO_3_^–^ influx. *Plant Physiol.* 93 1426–1432. 10.1104/pp.93.4.1426 16667635 PMC1062690

[B78] Šimura J. Antoniadi I. Široká J. Tarkowská D. Strnad M. Ljung K. (2018). Plant Hormonomics: Multiple Phytohormone Profiling by Targeted Metabolomics. *Plant Physiol.* 177 476–489. 10.1104/pp.18.00293 29703867 PMC6001343

[B79] Sinnott M. L. (1990). Catalytic mechanism of enzymic glycosyl transfer. *Chem. Rev.* 90 1171–1202. 10.1021/cr00105a006

[B80] Skinner W. Dahmani Z. Lu Y. Kridl J. C. Fazio G. C. (2012). A novel approach to measure crop plant protein expression. *LCGC Special Iss.* 10 16–21.

[B81] Snyman S. J. Hajari E. Watt M. P. Lu Y. Kridl J. C. (2015). Improved nitrogen use efficiency in transgenic sugarcane: phenotypic assessment in a pot trial under low nitrogen conditions. *Plant Cell Rep.* 34 667–669. 10.1007/s00299-015-1768-y 25686580

[B82] Solórzano L. (1969). Determination of ammonia in natural waters by the phenolhypochlorite method 1 1. This research was fully supported by U.S. Atomic Energy Commission Contract No. ATS (11-1) GEN 10, P.A. 20. *Limnol. Oceanogr.* 14 799–801. 10.4319/lo.1969.14.5.0799

[B83] Steinhauser D. Fernie A. R. Araújo W. L. (2012). Unusual cyanobacterial TCA cycles: not broken just different. *Trends Plant Sci.* 17 503–509. 10.1016/j.tplants.2012.05.005 22658681

[B84] Tingay S. Mcelroy D. Kalla R. Fieg S. Wang M. Thornton S. (1997). *Agrobacterium tumefaciens*-mediated barley transformation. *Plant J.* 11 1369–1376. 10.1046/j.1365-313X.1997.11061369.x

[B85] Tiong J. Mcdonald G. K. Genc Y. Pedas P. Hayes J. E. Toubia J. (2014). HvZIP7 mediates zinc accumulation in barley (Hordeum vulgare) at moderately high zinc supply. *N. Phytol.* 201 131–143. 10.1111/nph.12468 24033183

[B86] Tiwari J. K. Buckseth T. Devi S. Varshney S. Sahu S. Patil V. U. (2020a). Physiological and genome-wide RNA-sequencing analyses identify candidate genes in a nitrogen-use efficient potato cv. Kufri Gaurav. *Plant Physiol. Biochem.* 154 171–183. 10.1016/j.plaphy.2020.05.041 32563041

[B87] Tiwari J. K. Buckseth T. Zinta R. Saraswati A. Singh R. K. Rawat S. (2020b). Genome-wide identification and characterization of microRNAs by small RNA sequencing for low nitrogen stress in potato. *PLoS One* 15:233076. 10.1371/journal.pone.0233076 32428011 PMC7237020

[B88] Tiwari J. K. Buckseth T. Zinta R. Saraswati A. Singh R. K. Rawat S. (2020c). Transcriptome analysis of potato shoots, roots and stolons under nitrogen stress. *Sci. Rep.* 10:4. 10.1038/s41598-020-58167-4 31980689 PMC6981199

[B89] Trapnell C. Pachter L. Salzberg S. L. (2009). TopHat: discovering splice junctions with RNA-Seq. *Bioinformatics* 25 1105–1111. 10.1093/bioinformatics/btp120 19289445 PMC2672628

[B90] Ueguchi-Tanaka M. Ashikari M. Nakajima M. Itoh H. Katoh E. Kobayashi M. (2005). *GIBBERELLIN INSENSITIVE DWARF1* encodes a soluble receptor for gibberellin. *Nature* 437 693–698. 10.1038/nature04028 16193045

[B91] Vandesompele J. De Preter K. Pattyn F. Poppe B. Van Roy N. De Paepe A. (2002). Accurate normalization of real-time quantitative RT-PCR data by geometric averaging of multiple internal control genes. *Genome Biol.* 3 0031–0034. 10.1186/gb-2002-3-7-research0034 12184808 PMC126239

[B92] Vitousek P. M. Mooney H. A. Lubchenco J. Melillo J. M. (1997). Human domination of Earth’s ecosystems. *Science* 277 494–499. 10.1126/science.277.5325.494

[B93] Wang K. L. C. Li H. Ecker J. R. (2002). Ethylene biosynthesis and signaling networks. *Plant Cell* 14 S131–S151. 10.1105/tpc.001768 12045274 PMC151252

[B94] Wilson A. K. Latham J. R. Steinbrecher R. A. (2006). Transformation-induced Mutations in Transgenic Plants: Analysis and Biosafety Implications. *Biotechnol. Genet. Eng. Rev.* 23 209–238. 10.1080/02648725.2006.10648085 22530509

[B95] Yu J. L. Zhen X. X. Li X. Li N. Xu F. (2019). Increased Autophagy of Rice Can Increase Yield and Nitrogen Use Efficiency (NUE). *Front. Plant Sci.* 10:584. 10.3389/fpls.2019.00584 31134120 PMC6514234

[B96] Zhu Y. Nomura T. Xu Y. Zhang Y. Peng Y. Mao B. (2006). *ELONGATED UPPERMOST INTERNODE* encodes a cytochrome P450 monooxygenase that epoxidizes gibberellins in a novel deactivation reaction in rice. *Plant Cell* 18 442–456. 10.1105/tpc.105.038455 16399803 PMC1356550

