# Supplementary figures and images for: Improving Nitrogen Use Efficiency Through Overexpression of Alanine Aminotransferase in Rice, Wheat, and Barley
Source: Front Plant Sci. 2021 Jan 28;12:628521. doi: 10.3389/fpls.2021.628521 (PMC7875890; doi:10.3389/fpls.2021.628521)

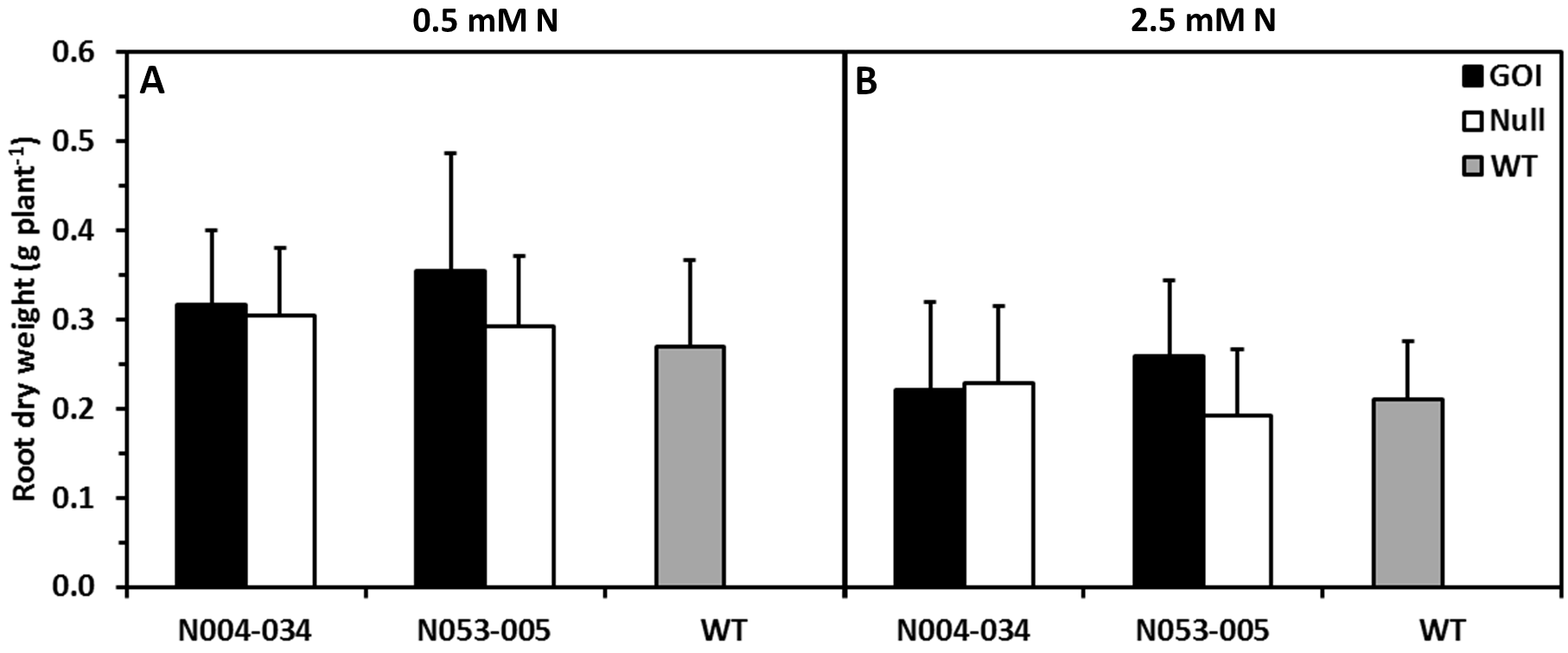

Supplement: Supplementary Figure 1 — Effects of low and adequate N treatment on root biomass of rice plants expressing OsAnt1:HvAlaAT. [file Data_Sheet_1.ZIP › Figure S1.tif]

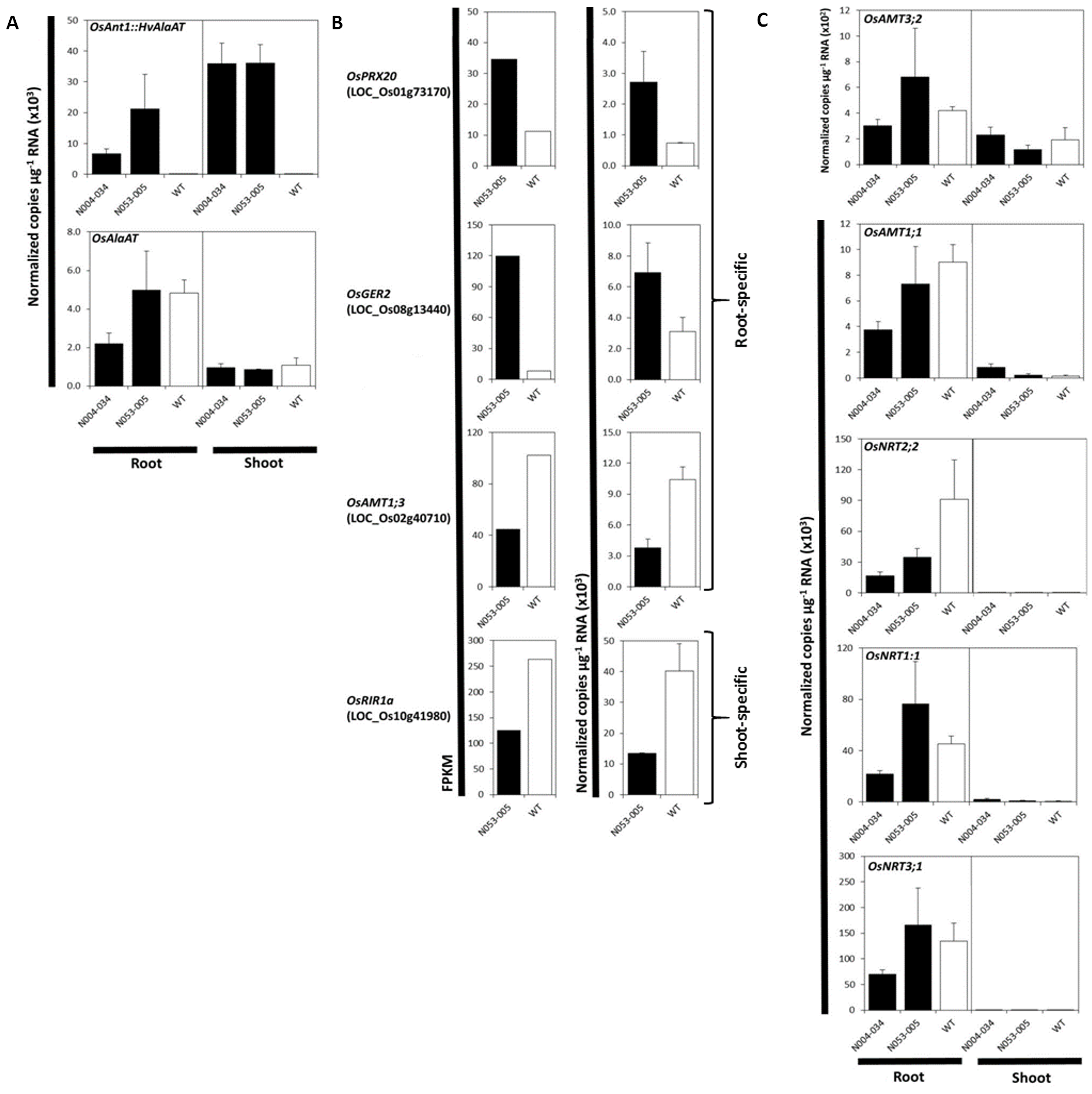

Supplement: Supplementary Figure 1 — Effects of low and adequate N treatment on root biomass of rice plants expressing OsAnt1:HvAlaAT. [file Data_Sheet_1.ZIP › Figure S10.tif]

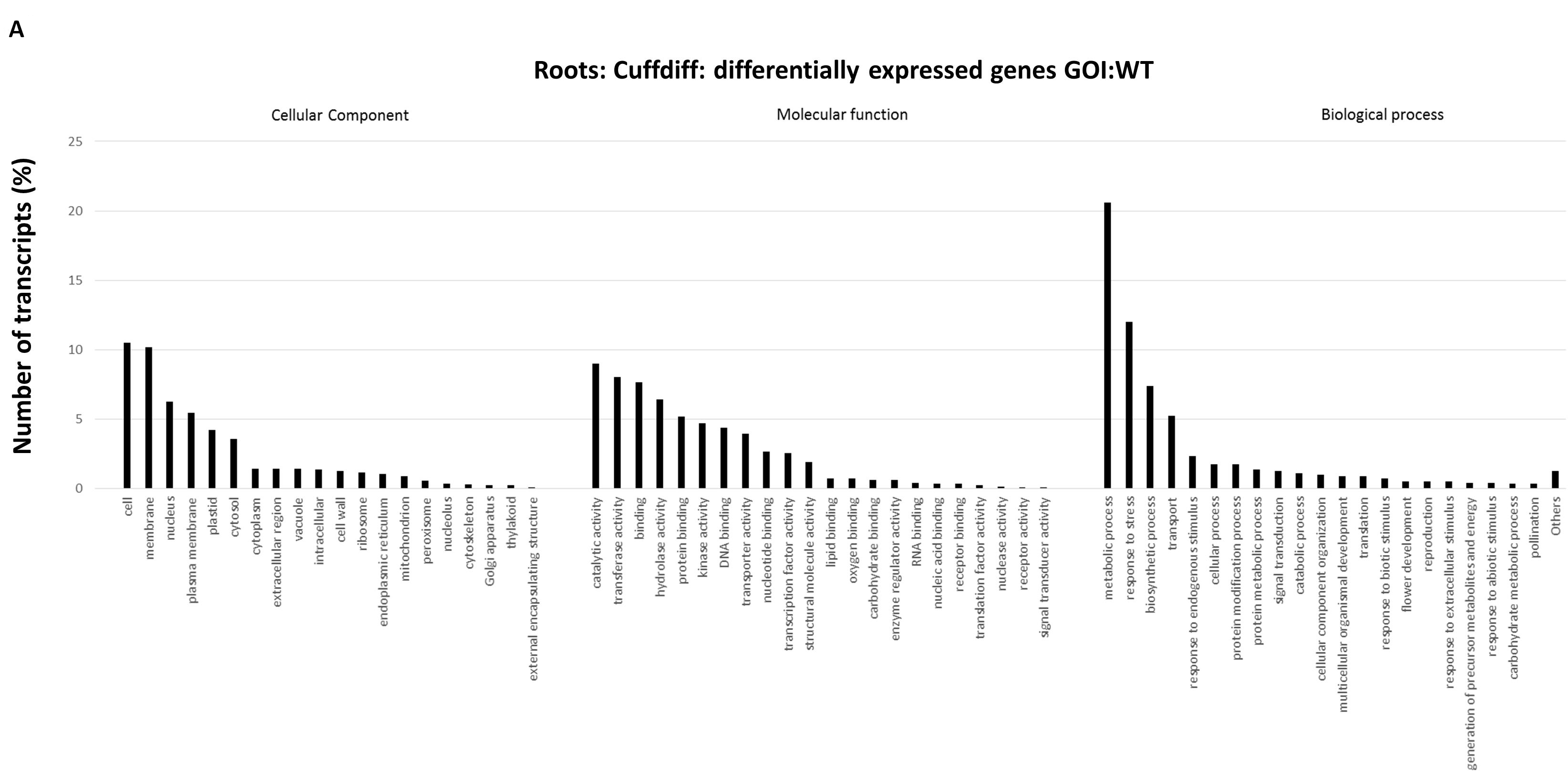

Supplement: Supplementary Figure 1 — Effects of low and adequate N treatment on root biomass of rice plants expressing OsAnt1:HvAlaAT. [file Data_Sheet_1.ZIP › Figure S11A.tif]

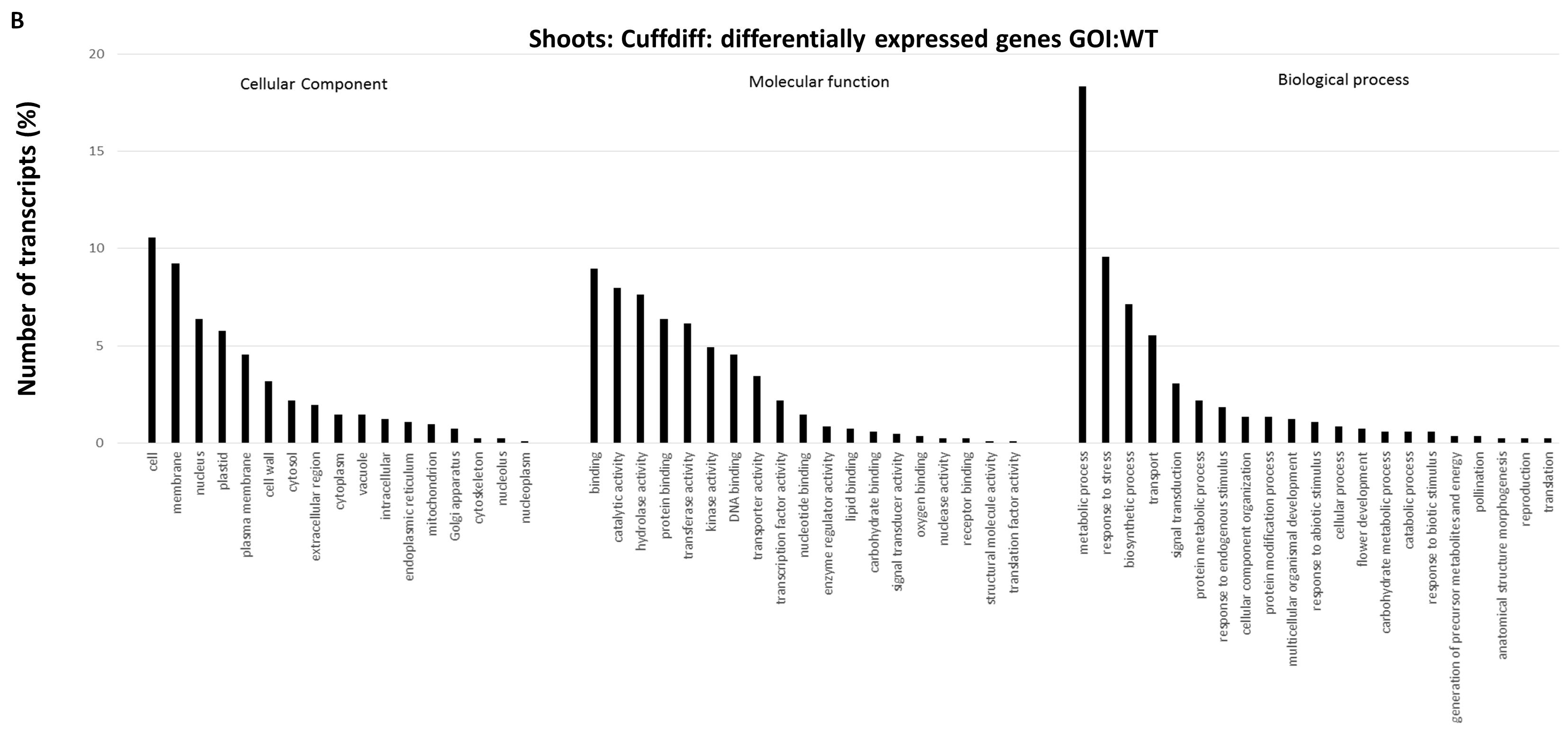

Supplement: Supplementary Figure 1 — Effects of low and adequate N treatment on root biomass of rice plants expressing OsAnt1:HvAlaAT. [file Data_Sheet_1.ZIP › Figure S11B.tif]

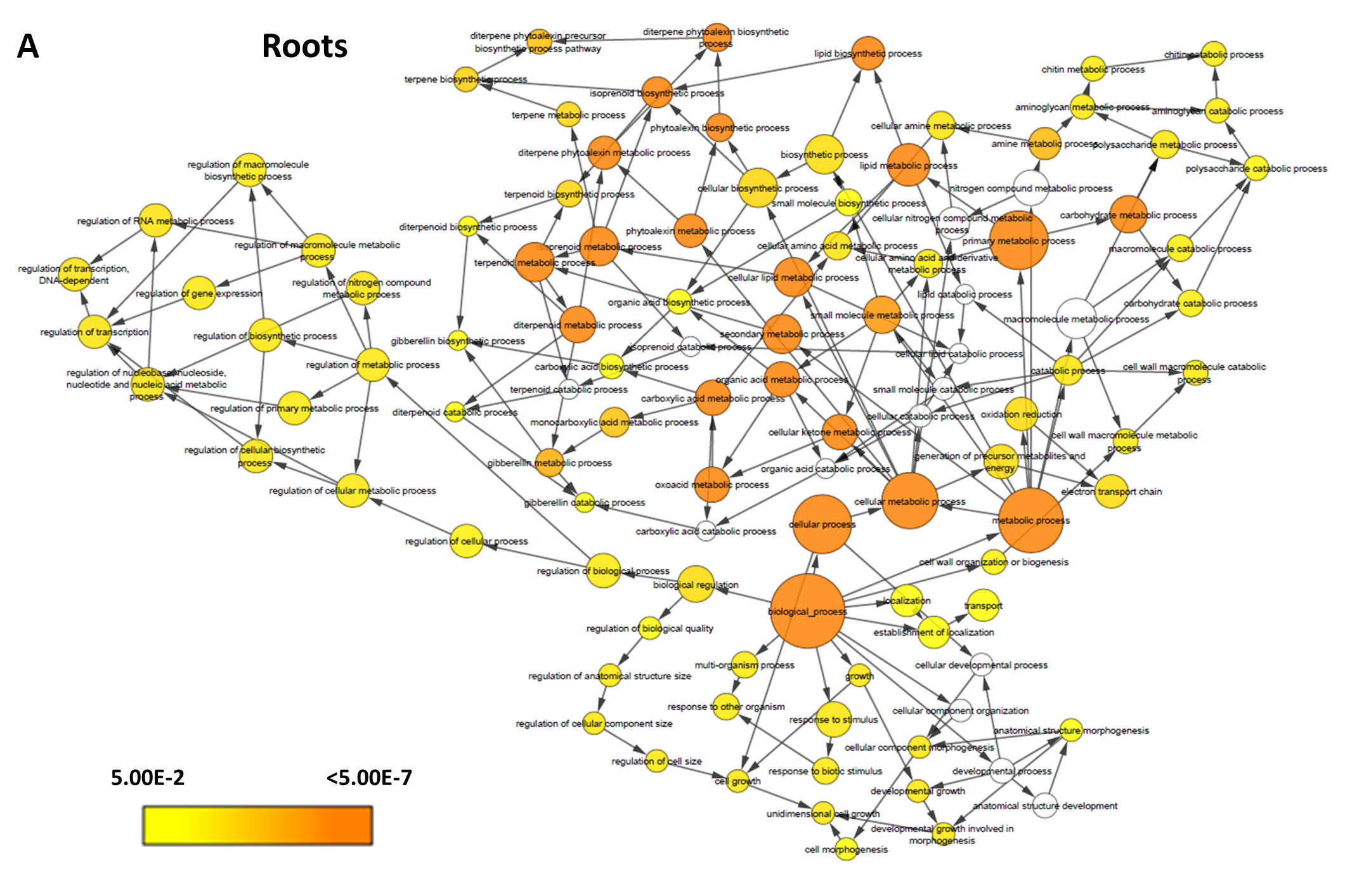

Supplement: Supplementary Figure 1 — Effects of low and adequate N treatment on root biomass of rice plants expressing OsAnt1:HvAlaAT. [file Data_Sheet_1.ZIP › Figure S12A.tif]

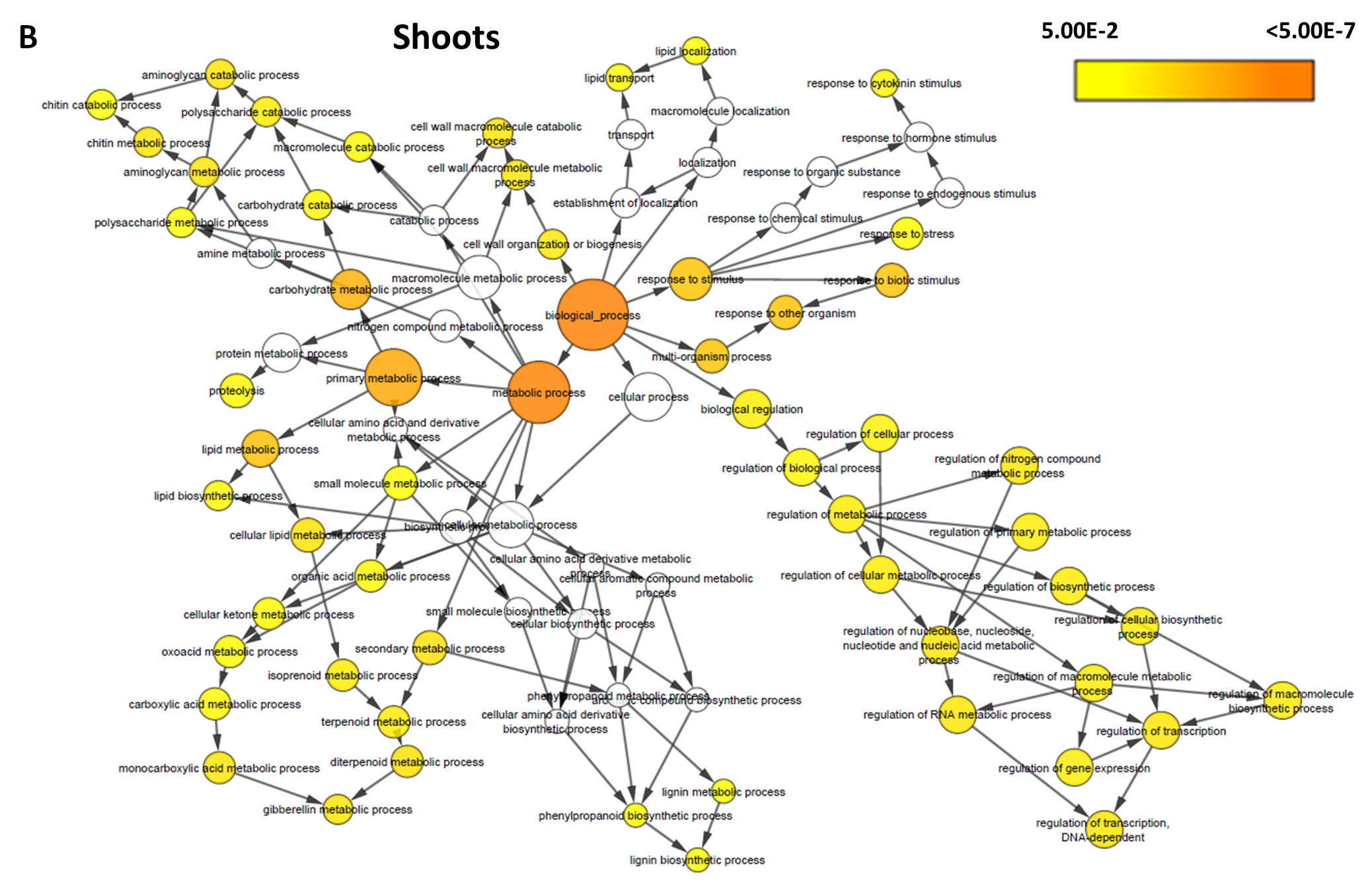

Supplement: Supplementary Figure 1 — Effects of low and adequate N treatment on root biomass of rice plants expressing OsAnt1:HvAlaAT. [file Data_Sheet_1.ZIP › Figure S12B.tif]

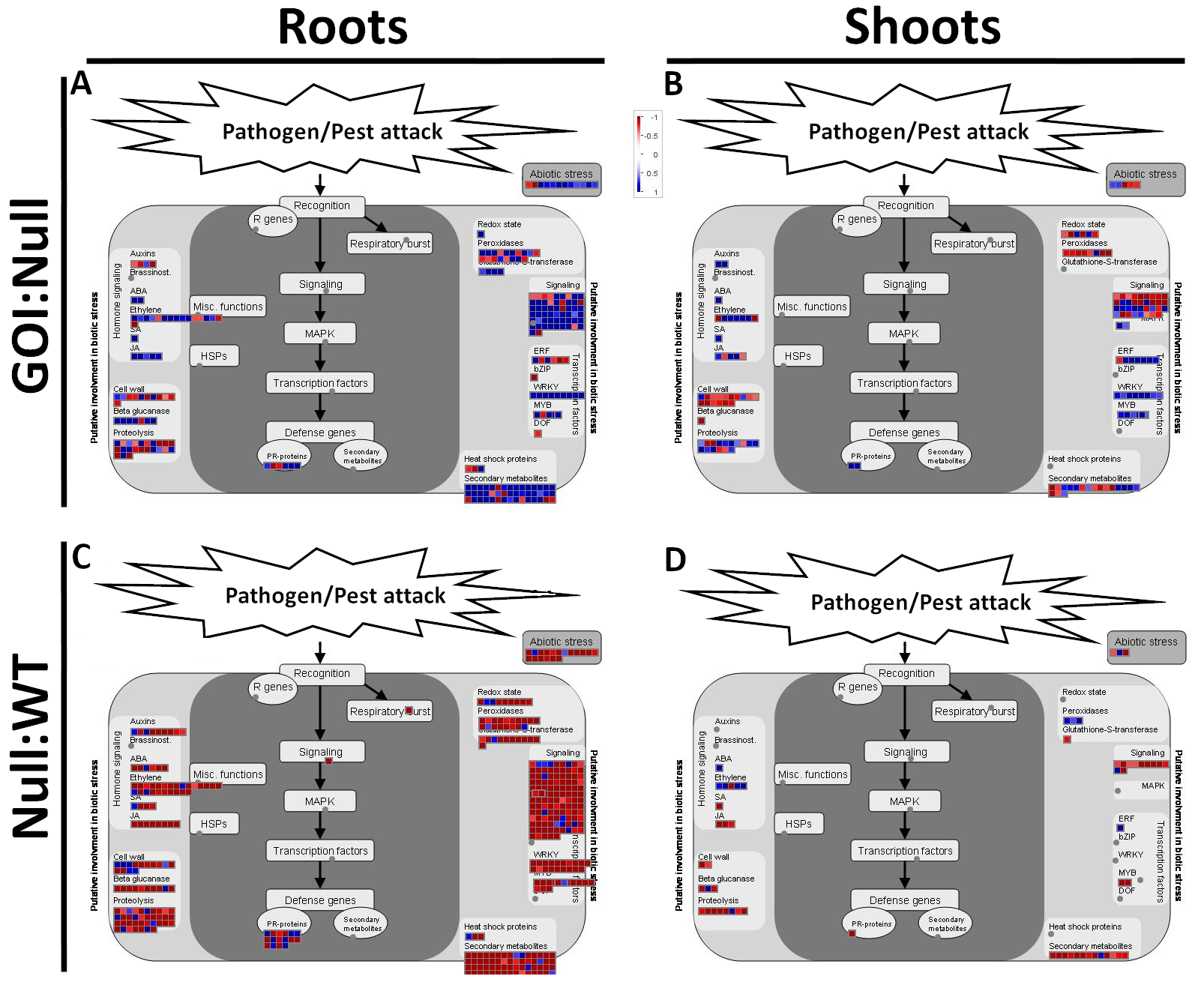

Supplement: Supplementary Figure 1 — Effects of low and adequate N treatment on root biomass of rice plants expressing OsAnt1:HvAlaAT. [file Data_Sheet_1.ZIP › Figure S13.tif]

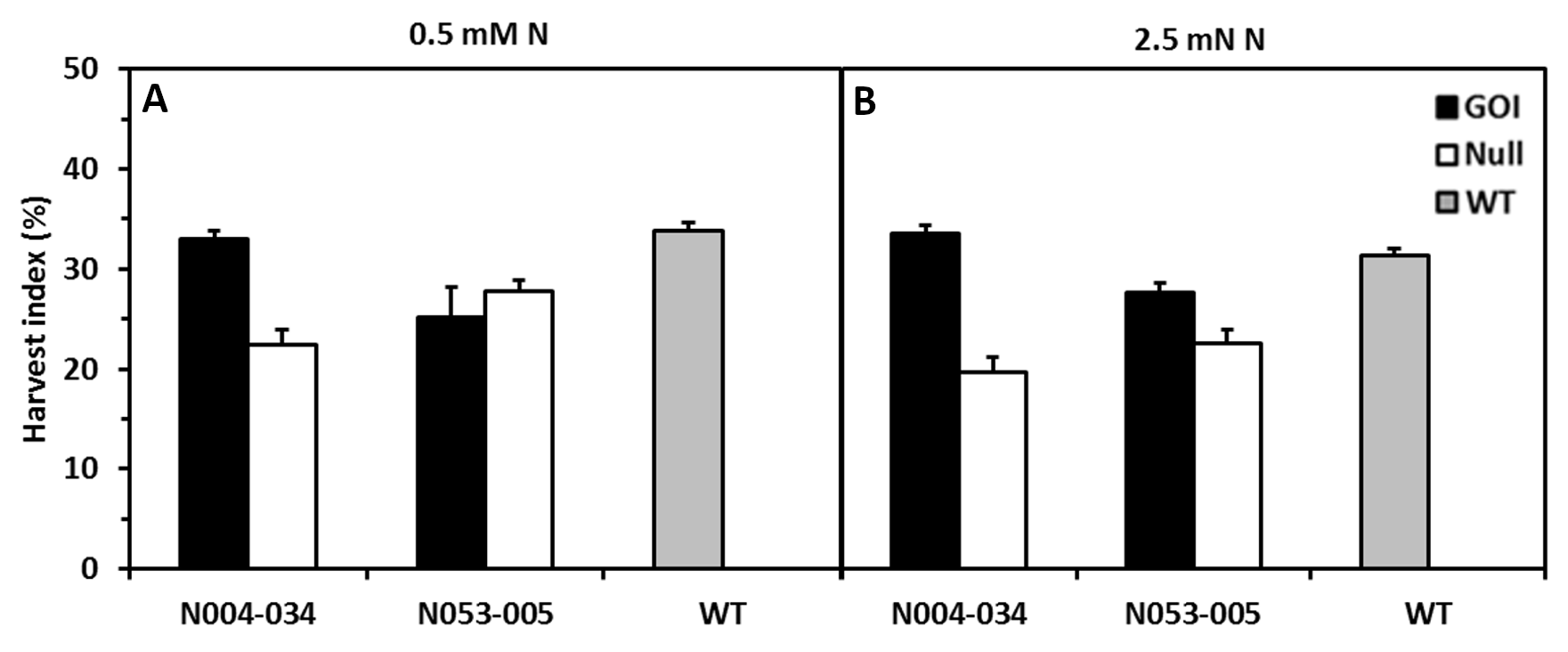

Supplement: Supplementary Figure 1 — Effects of low and adequate N treatment on root biomass of rice plants expressing OsAnt1:HvAlaAT. [file Data_Sheet_1.ZIP › Figure S2.tif]

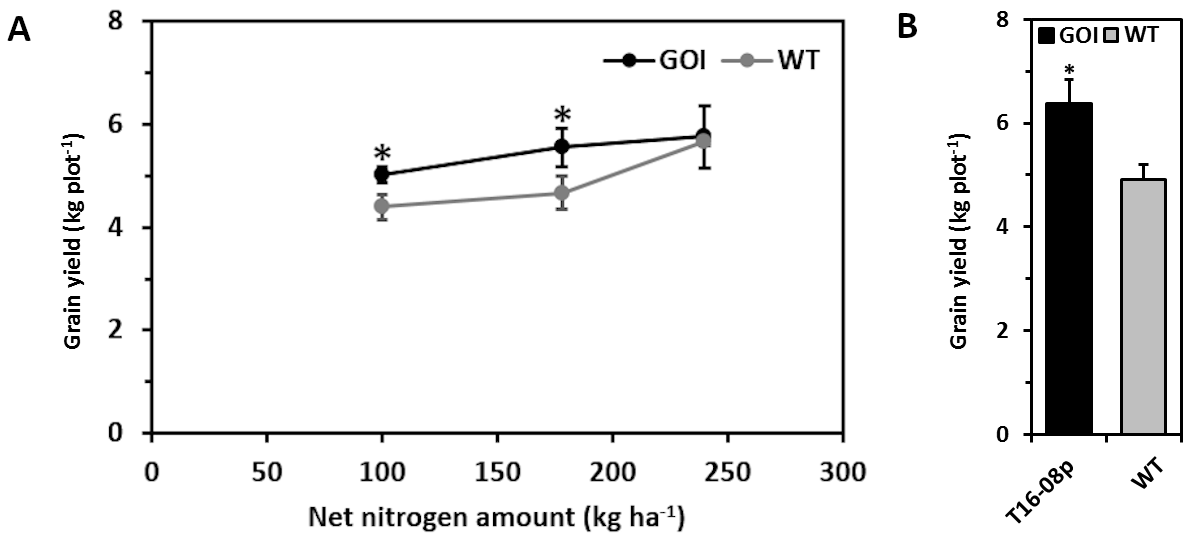

Supplement: Supplementary Figure 1 — Effects of low and adequate N treatment on root biomass of rice plants expressing OsAnt1:HvAlaAT. [file Data_Sheet_1.ZIP › Figure S3.tif]

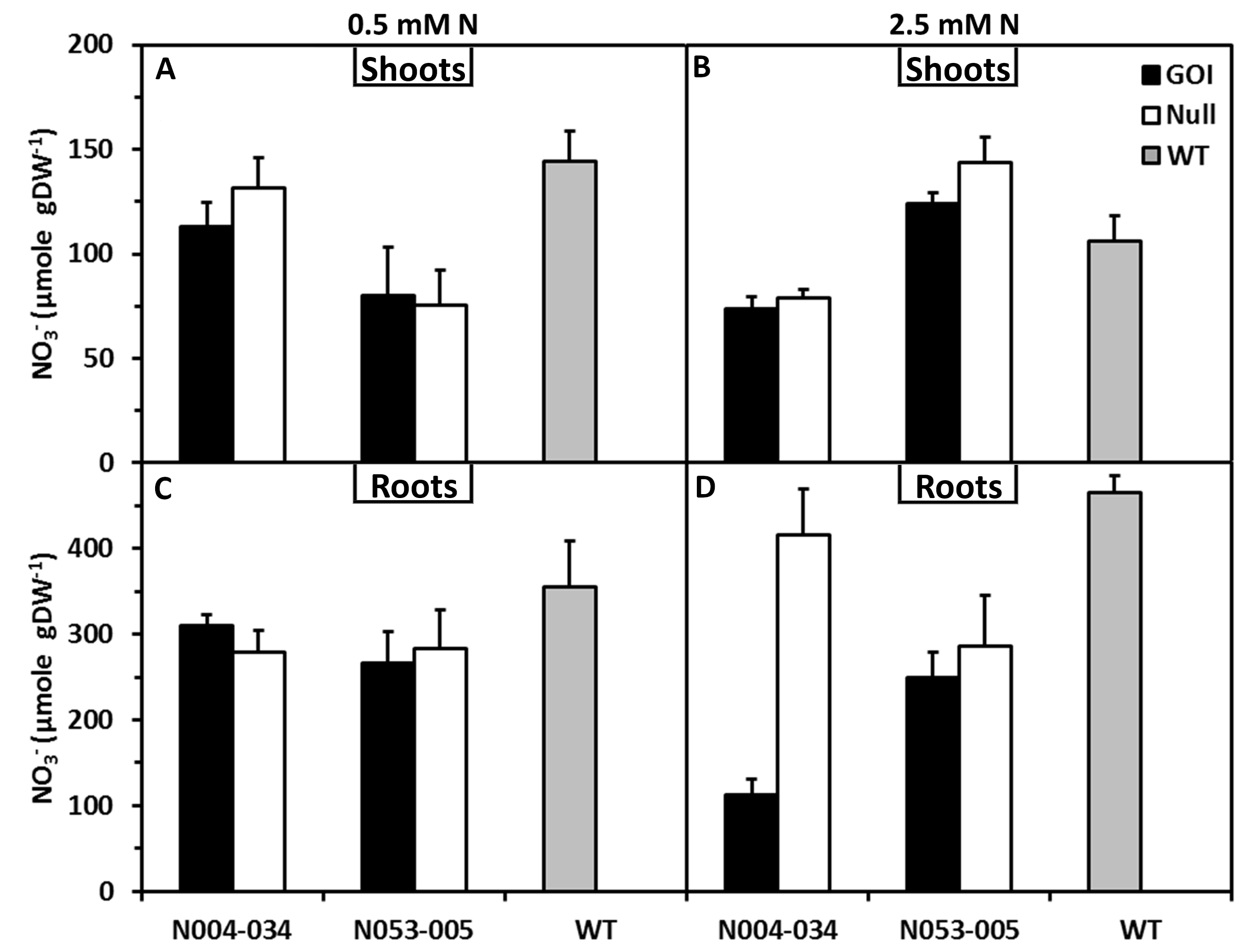

Supplement: Supplementary Figure 1 — Effects of low and adequate N treatment on root biomass of rice plants expressing OsAnt1:HvAlaAT. [file Data_Sheet_1.ZIP › Figure S4.tif]

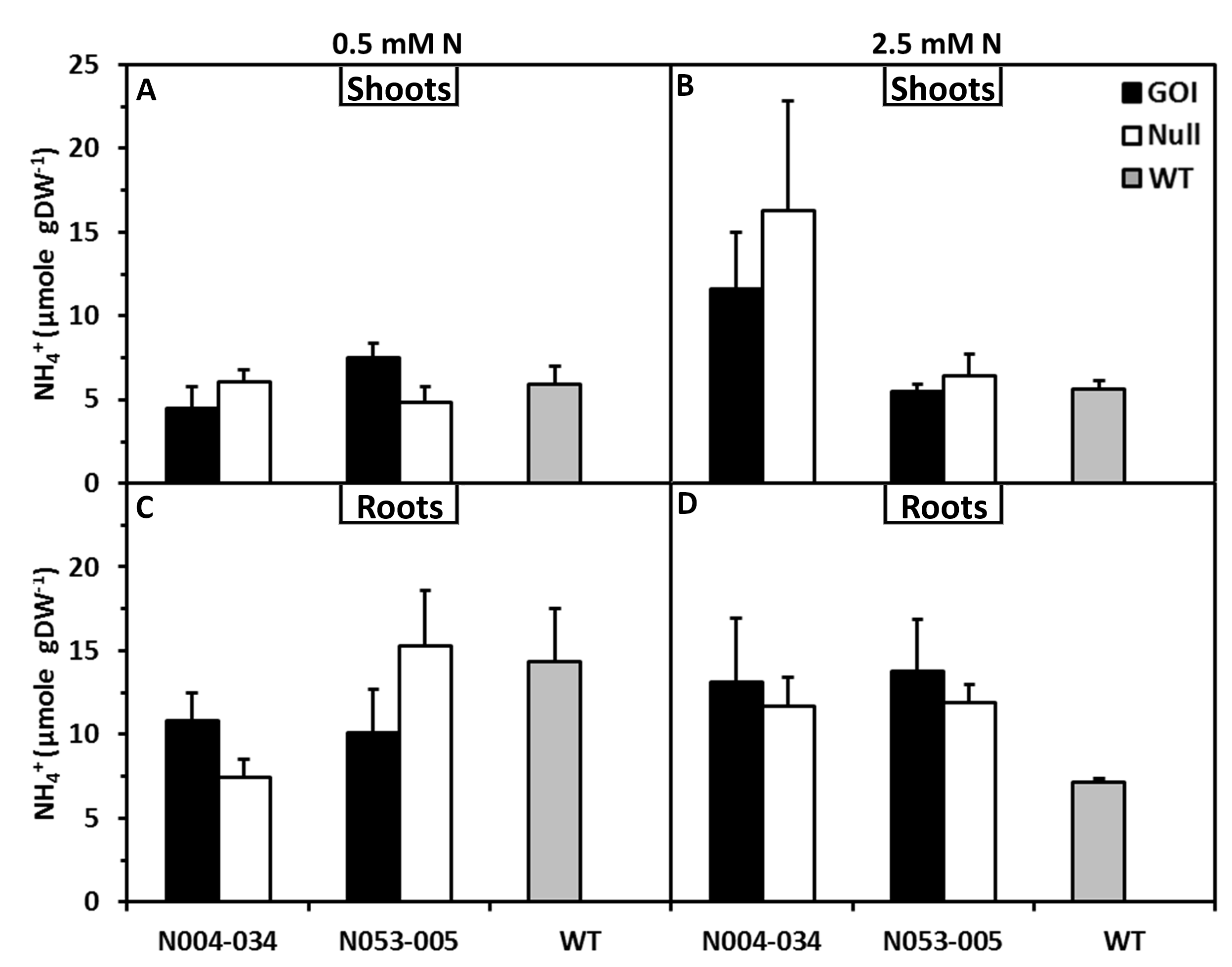

Supplement: Supplementary Figure 1 — Effects of low and adequate N treatment on root biomass of rice plants expressing OsAnt1:HvAlaAT. [file Data_Sheet_1.ZIP › Figure S5.tif]

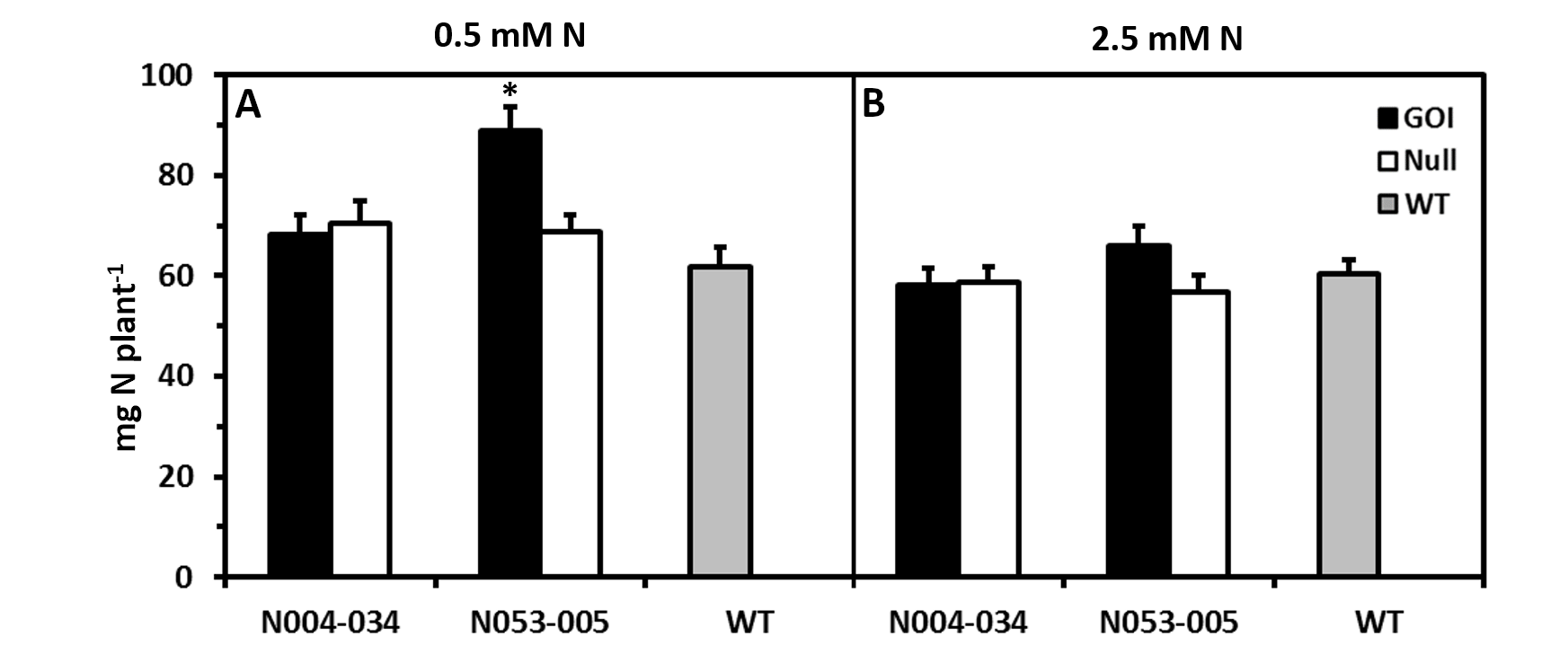

Supplement: Supplementary Figure 1 — Effects of low and adequate N treatment on root biomass of rice plants expressing OsAnt1:HvAlaAT. [file Data_Sheet_1.ZIP › Figure S6.tif]

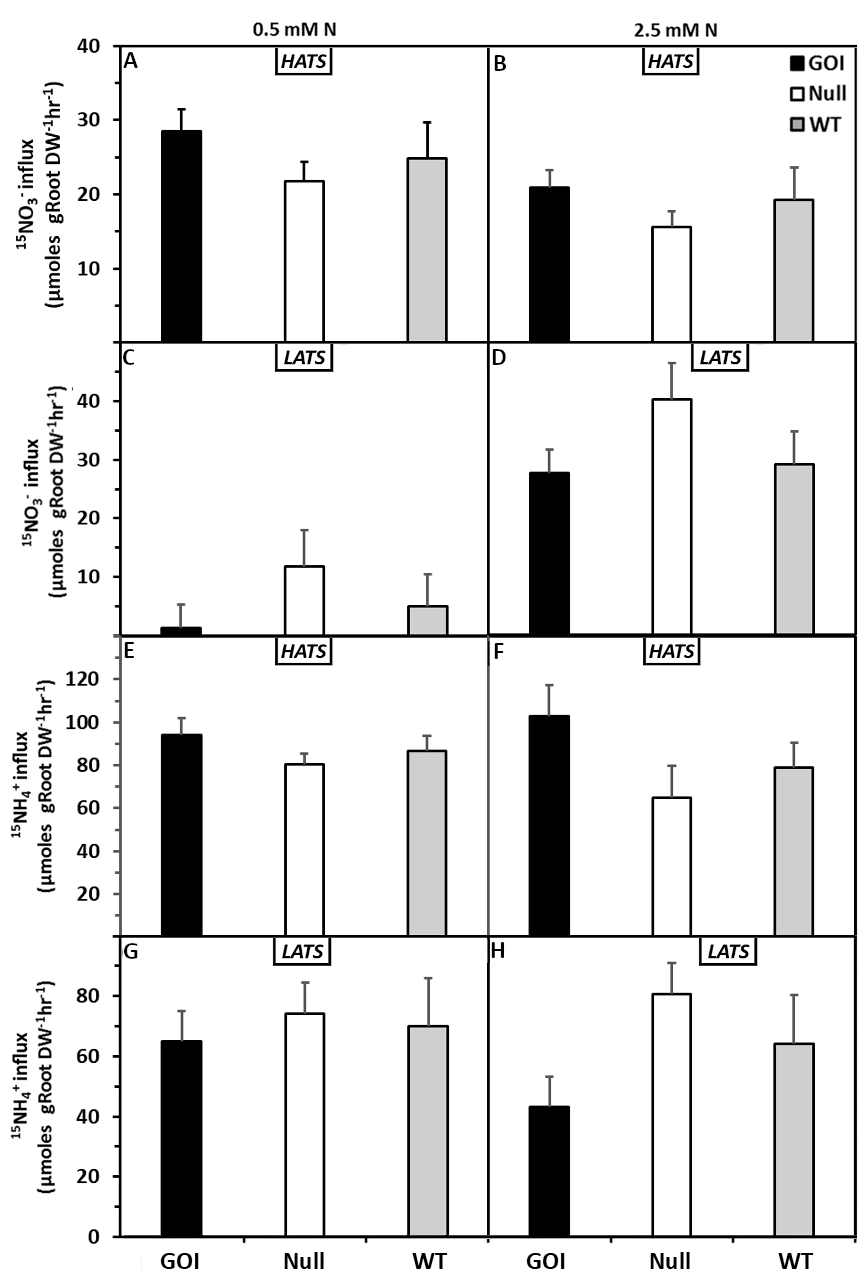

Supplement: Supplementary Figure 1 — Effects of low and adequate N treatment on root biomass of rice plants expressing OsAnt1:HvAlaAT. [file Data_Sheet_1.ZIP › Figure S7.tif]

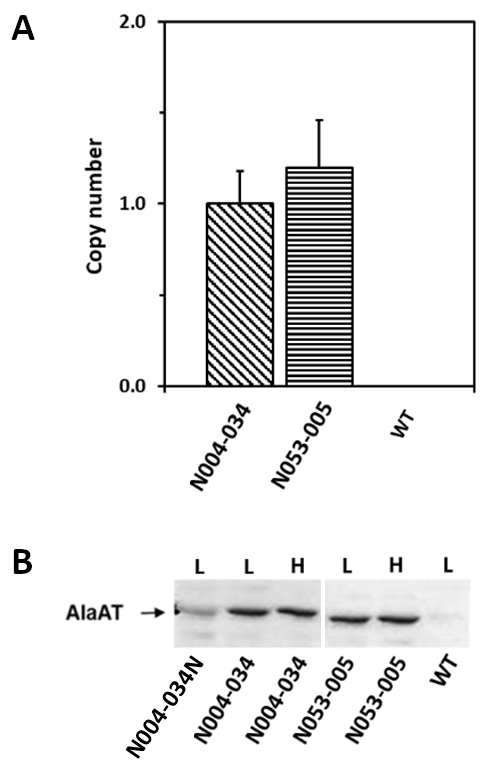

Supplement: Supplementary Figure 1 — Effects of low and adequate N treatment on root biomass of rice plants expressing OsAnt1:HvAlaAT. [file Data_Sheet_1.ZIP › Figure S8.tif]

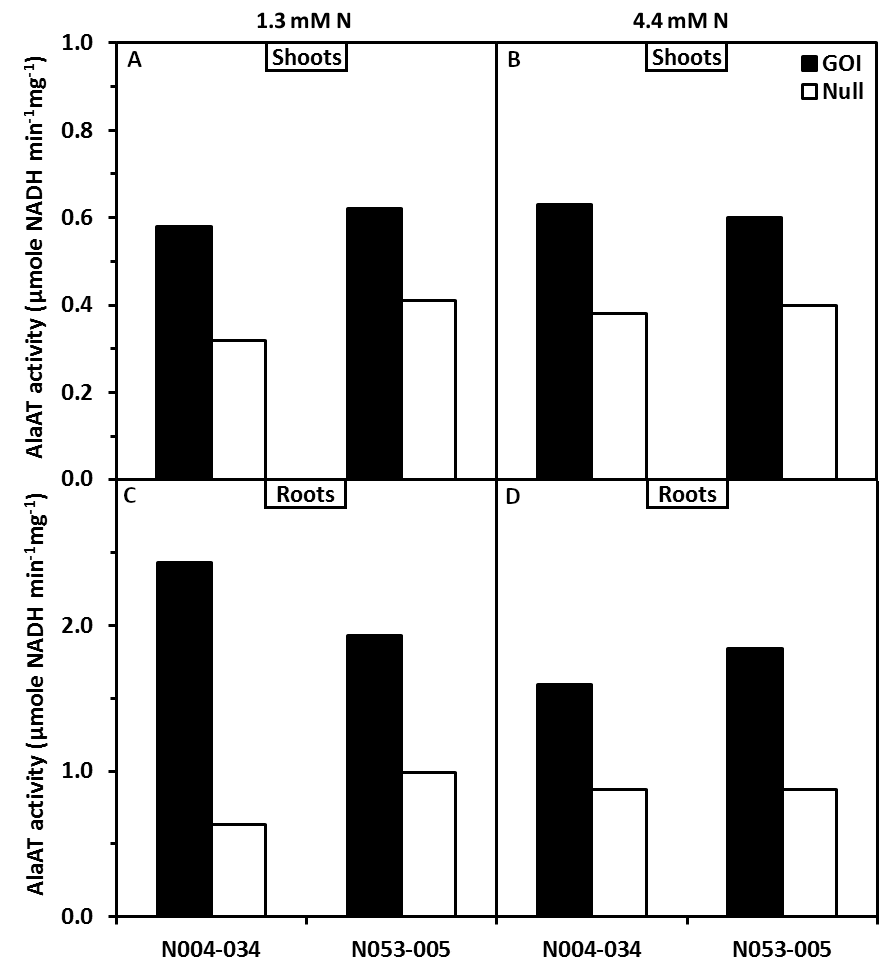

Supplement: Supplementary Figure 1 — Effects of low and adequate N treatment on root biomass of rice plants expressing OsAnt1:HvAlaAT. [file Data_Sheet_1.ZIP › Figure S9.tif]
