# Supplementary material for: Improving Nitrogen Use Efficiency Through Overexpression of Alanine Aminotransferase in Rice, Wheat, and Barley
Source: Front Plant Sci. 2021 Jan 28;12:628521. doi: 10.3389/fpls.2021.628521 (PMC7875890; doi:10.3389/fpls.2021.628521)
Supplement: Supplementary Method — AlaAT protein expression and enzyme activity. [file Data_Sheet_2.docx]

**Supplementary Method**

***AlaAT protein expression and enzyme activity***

Three-inch tips of young leaves at maximum tillering were collected and stored in RNAlater^®^ (Qiagen). The plants were grown in a field with 90kg N.ha^-1^, which was similar to the trial described in the text. Tissues were pooled by genotype for simultaneous isolation of RNA and protein using NucleoSpin RNA/Protein Kits (Macherey-Nagel, Bethlehem, PA).

Protein samples (15µg) were used for a Western blot using HvAlaAT rabbit polyclonal antibodies. Proteins were separated on a NuPAGE 10% Bis-Tris gel (Invitrogen) with 1x MOPS SDS running buffer (Invitrogen) and transferred to a nitrocellulose membrane using an iBlot dry blotting system (Invitrogen). Membranes were blocked, hybridized with the antibody at 1:5000 overnight at 4°C, washed and hybridized to horseradish peroxidase-conjugated secondary antibody. Membranes were developed using SuperSignal West Pico Chemiluminescent substrate kit (Thermofisher Scientific).

Protein for enzyme assay was isolated from four young leaves or primary roots, pooled from two plants each, grown in a hydroponic system with either low (1.3mM) or high (4.4mM) N supplied as 25% NH_4_NO_3_ and 75% Ca(NO_3_)_2_. Hydroponic tanks held 96 plants and eight litres of nutrient solution based on Yoshida et al. ([1971](#_ENREF_2)) with solutions changed weekly. Tissue from 46-day-old plants (250mg) was ground in 2ml extraction buffer, and 25µl of supernatant was assayed spectrophotometrically in a coupled enzyme reaction with lactate dehydrogenase and NADH as described previously ([Muench and Good, 1994](#_ENREF_1)) using 2-oxoglutarate and alanine as substrates. Protein was quantified using the Bradford method (Sigma Aldrich).

Muench, D.G., and Good, A.G. (1994). Hypoxically inducible barley alanine aminotransferase: cDNA cloning and expression analysis. *Plant Mol. Biol.* 24**,** 417-427. doi: 10.1007/BF00024110

Yoshida, S., Forno, D.A., and Cock, J.H. (1971). *Laboratory manual for physiological studies of rice.* IRRI, Los Baños, Philippines.
